# Validation of Novel Prognostic Biomarkers for Early-Stage Clear-Cell, Endometrioid and Mucinous Ovarian Carcinomas Using Immunohistochemistry

**DOI:** 10.3389/fonc.2020.00162

**Published:** 2020-02-18

**Authors:** Hanna Engqvist, Toshima Z. Parris, Anikó Kovács, Elisabeth Werner Rönnerman, Karin Sundfeldt, Per Karlsson, Khalil Helou

**Affiliations:** ^1^Department of Oncology, Sahlgrenska Cancer Center, Institute of Clinical Sciences, Sahlgrenska Academy at University of Gothenburg, Gothenburg, Sweden; ^2^Department of Clinical Pathology, Sahlgrenska University Hospital, Gothenburg, Sweden; ^3^Department of Obstetrics and Gynecology, Sahlgrenska Cancer Center, Institute of Clinical Sciences, Sahlgrenska Academy at University of Gothenburg, Gothenburg, Sweden

**Keywords:** clear-cell ovarian carcinoma, endometrioid ovarian carcinoma, mucinous ovarian carcinoma, histotype-specific prognostic biomarkers, immunohistochemistry, early-stage ovarian carcinoma

## Abstract

Early-stage (I and II) ovarian carcinoma patients generally have good prognosis. Yet, some patients die earlier than expected. Thus, it is important to stratify early-stage patients into risk groups to identify those in need of more aggressive treatment regimens. The prognostic value of 29 histotype-specific biomarkers identified using RNA sequencing was evaluated for early-stage clear-cell (CCC), endometrioid (EC) and mucinous (MC) ovarian carcinomas (*n* = 112) using immunohistochemistry on tissue microarrays. Biomarkers with prognostic significance were further evaluated in an external ovarian carcinoma data set using the web-based Kaplan-Meier plotter tool. Here, we provide evidence of aberrant protein expression patterns and prognostic significance of 17 novel histotype-specific prognostic biomarkers [10 for CCC (ARPC2, CCT5, GNB1, KCTD10, NUP155, RPL13A, RPL37, SETD3, SMYD2, TRIO), three for EC (CECR1, KIF26B, PIK3CA), and four for MC (CHEK1, FOXM1, KIF23, PARPBP)], suggesting biological heterogeneity within the histotypes. Combined predictive models comprising the protein expression status of the validated CCC, EC and MC biomarkers together with established clinical markers (age, stage, CA125, ploidy) improved the predictive power in comparison with models containing established clinical markers alone, further strengthening the importance of the biomarkers in ovarian carcinoma. Further, even improved predictive powers were demonstrated when combining these models with our previously identified prognostic biomarkers PITHD1 (CCC) and GPR158 (MC). Moreover, the proteins demonstrated improved risk prediction of CCC-, EC-, and MC-associated ovarian carcinoma survival. The novel histotype-specific prognostic biomarkers may not only improve prognostication and patient stratification of early-stage ovarian carcinomas, but may also guide future clinical therapy decisions.

## Introduction

If diagnosed early, epithelial ovarian carcinoma patients have a relatively good prognosis with an overall 5-year survival rate of 89% for stage I and 71% for stage II (1). Unfortunately, around 16% of early-stage ovarian carcinoma patients are at greater risk of relapse and early death. Hence, the identification of molecular tumor characteristics associated with high-risk early-stage ovarian carcinomas would improve risk assessment, potentially influence treatment decisions, and guide future drug development. Recently, various studies have shown the importance of histotype-based stratification in view of differences in e.g., molecular and clinical behavior, and prognosis with significant differences in 5-year survival rates across histotypes [43% for serous ovarian carcinoma (SC), high-grade serous (HGSC), and low grade serous ovarian carcinoma (LGSC), 82% for EC, 71% for MC, and 66% for CCC] (1). Thus, it is crucial to evaluate prognostication within individual histotypes to identify early molecular events of histotype-specific tumorigenesis. To date, limited information is available for prognostic biomarkers associated with specific histotypes and early-stage disease.

In recent years, a number of studies have evaluated the prognostic significance of specific biomarkers within ovarian cancer histotypes. The prognostic role of p16 was examined in a large cohort of ovarian carcinoma patients (*n* = 6,525), wherein differences in prognosis were demonstrated across the five main histotypes of varying FIGO stages. Block expression (overexpression >90% of tumor cells are stained) of p16 was associated with shorter overall survival (OS) in CCC and EC, absence of p16 in LGSC correlated with shorter OS, while no prognostic significance was found for HGSC- or MC-patients (2). A further study showed an association between favorable outcome and ARID1A- and p53-expression, as well as negative nuclear/positive membrane expression for β-Catenin, in 97 ovarian [CCC (*n* = 11), EC (*n* = 21)] and endometrial [clear-cell (*n* = 6) and endometrioid uterine (*n* = 59)] carcinomas. However, prognosis was investigated in all 97 patient samples regardless of type of carcinoma, histotype or FIGO stages I–IV (3). A recent study examined the prognostic role of the five main histotypes in early-stage ovarian carcinomas (*n* = 488), wherein EC was found to be the most favorable histotype, while HGSC and LGSC had the most unfavorable prognoses. Further, CCC with abnormal p53 protein staining patterns was also reported to have poor prognosis (4). Moreover, patients with stage Ia or Ib of EC or MC histotypes have been shown to have a 10-year disease-specific survival over 95% (5).

Therefore, reliable early-stage histotype-specific biomarkers that are independent and complementary to established clinical markers are needed to improve future prognostication at the time of diagnosis, risk stratification and the administration of adequate drugs for early-stage ovarian carcinoma patients. Here, we used immunohistochemistry (IHC) on tissue microarrays (TMA) to examine the prognostic role of 29 previously identified RNA-based biomarkers for histotype-specific, early-stage ovarian carcinoma [11 biomarkers associated with CCC (ARPC2, CCT5, DDX24, GNB1, KCTD10, NUP155, RPL13A, RPL37, SETD3, SMYD2, TRIO), eight with EC (ABCA12, CECR1, ESRRG, KIF26B, MUC15, PDE4DIP, PIK3CA, RIMBP2), and 10 with MC (CENPI, CHEK1, FOXM1, KIF15, KIF23, KNTC1, MTGR1, NSD2, PARPBP, ZDHHC2)].

## Materials and Methods

### Patients and Tissue Microarray Construction

The patient study cohort comprised 112 early-stage (stage I and II) primary invasive ovarian carcinoma patients (diagnosed between 1994 and 2006) of histotypes clear cell carcinoma [CCC (*n* = 37)], endometrioid carcinoma [EC (*n* = 46)] and mucinous carcinoma [MC (*n* = 29)]. Full face formalin-fixed paraffin-embedded (FFPE) specimens corresponding to the 112 patients were reclassified in 2016 by board certified pathologists at Sahlgrenska University Hospital according to current WHO criteria for ovarian carcinoma histotypes (6). The clinicopathological information, obtained from the National Quality Registry at the Regional Cancer Center West (Gothenburg, Sweden) and the Cancer Registry at the National Board of Health and Welfare (Stockholm, Sweden), is summarized in Table 1. The FFPE specimens were obtained from the Departments of Clinical Pathology at hospitals in Western Sweden in accordance with the Declaration of Helsinki and approved by the Regional Ethical Review Board (case number 767-14, Gothenburg, Sweden). The ethical review board further approved a waiver of written consent to use the tumor specimens.

**Table 1:** Clinicopathological data for the patient cohort (*n* = 112) comprising clear-cell (CCC), endometrioid (EC) and mucinous ovarian carcinoma (MC) histotypes.

	**Number of patients (%)**			
	**CCC (*n* = 37)**	**EC (*n* = 46)**	**MC (*n* = 29)**	***P*-value**
Patient age				0.37
Mean	65	62	60	
Range	42–84	25–83	30–82	
Overall survival				0.24
0–2 y	5 (14)	3 (7)	6 (21)	
2–5 y	10 (27)	9 (20)	3 (10)	
5–10 y	8 (22)	7 (15)	7 (24)	
>10 y	14 (38)	27 (59)	13 (45)	
Cause of death				**0.023**
Ovarian carcinoma	19 (51)	7 (15)	5 (17)	
Other cancer	2 (5)	6 (13)	4 (14)	
Other	6 (16)	10 (22)	8 (28)	
Alive	8 (22)	17 (37)	7 (24)	
Not available	2 (5)	6 (13)	5 (17)	
Stage				0.43
I	31 (84)	32 (70)	22 (76)	
II	6 (16)	13 (28)	7 (24)	
Tumor grade EC				NA
FIGO grade I	NA	11 (24)	NA	
FIGO grade II	NA	27 (59)	NA	
FIGO grade III	NA	8 (17)	NA	
CA125				0.58
<35	14 (38)	13 (28)	10 (35)	
35–65	8 (22)	7 (15)	8 (28)	
>65	15 (41)	25 (54)	11 (38)	
Not available	0 (0)	1 (2)	0 (0)	
Ploidy				0.14
Near diploid	5 (14)	17 (37)	7 (24)	
Aneuploid	30 (81)	26 (57)	19 (66)	
Not available	2 (5)	3 (7)	3 (10)	
Chemotherapy				0.20
Yes	37 (100)	42 (91)	27 (93)	
No	0 (0)	0 (0)	0 (0)	
Not available	0 (0)	4 (9)	2 (7)	

*Significant values (P-value < 0.05) are marked in bold*.

Prior to TMA construction, tumor areas were marked on a hematoxylin and eosin stained slide for each tumor sample. TMAs were prepared comprising 1.0 mm triplicate cores from each tumor with 1.6 mm spacing distance between core centers. The TMA block was baked for 1 h at 45°C. Four micrometer TMA sections were processed on microscope slides (FLEX IHC, Dako, Sweden) and dried in an oven for 1 h at 60°C.

### Selection of Genes Associated With Histotype-Specific Prognosis

The retrieval of prognostic genetic signatures for overall survival (OS) and disease-specific survival (DSS) using 45/112 raw RNA sequencing (RNA-seq) read counts for CCC, EC, and MC histotypes and univariable Cox regression models has been described elsewhere (7). The predictive performance of the Cox regression models was measured using concordance index (C-index), with values ranging from 0.5 to 1, wherein 1 is a perfect prediction of survival outcome (8). Among the top 50 genes (*P*-values < 0.05 and C-index >0.75) for each respective histotype, 29 genes were selected among those with gene expression levels which could be measured using IHC (RNA-seq counts >150) (Table 2).

**Table 2:** Study cohort with respective statistical features derived from Cox proportional hazard models and selected antibodies with corresponding optimized antibody dilution factors for immunohistochemistry (IHC) analysis.

**Gene symbol**	**Histotype**	**Survival**	**HR**	**95% CI**	***P-*value**	**C-index**	**Antibody**	**Company**	**Optimized dilution**
*ABCA12*	EC	DSS	0.50	0.28–0.89	0.019	0.90	ab98976	Abcam	1:25
*ARPC2*	CCC	OS	17.48	2.95–103.41	0.0016	0.79	HPA008352	Sigma-Aldrich	1:200
*CCT5*	CCC	DSS	17.81	2.42–131.34	0.0047	0.83	H00022948	Abnova	1:2,000
*CECR1*	EC	OS	0.99	0.98–1.0	0.0110	0.76	SAB1410953	Sigma-Aldrich	1:25
*CENPI*	MC	OS	4.68	1.40–15.68	0.0120	0.85	ab118796	Abcam	1:100
*CHEK1*	MC	OS	4.11	1.39–12.16	0.0110	0.85	AV32589	Sigma-Aldrich	1:25
*DDX24*	CCC	OS	22.87	3.35–156.11	0.0014	0.78	HPA002554	Sigma-Aldrich	1:25
*ESRRG*	EC	DSS	0.59	0.35–0.98	0.0420	0.89	AV31655	Sigma-Aldrich	1:100
*FOXM1*	MC	OS	2.55	1.17–5.55	0.0180	0.83	HPA029974	Sigma-Aldrich	1:100
*GNB1*	CCC	OS	66.42	5.35–824.60	0.0011	0.81	SAB2701168	Sigma-Aldrich	1:250
*KCTD10*	CCC	OS	42.42	4.88–368.71	0.00068	0.81	ab129245	Abcam	1:30
*KIF15*	MC	OS	4.69	1.15–19.03	0.0310	0.84	HPA035517	Sigma-Aldrich	1:25
*KIF23*	MC	OS	7.88	1.33–46.52	0.0230	0.85	SAB2104085	Sigma-Aldrich	1:50
*KIF26B*	EC	OS	0.46	0.23–0.91	0.0250	0.91	HPA028562	Sigma-Aldrich	1:25
*KNTC1*	MC	OS	8.46	1.46–48.93	0.017	0.83	HPA025241	Sigma-Aldrich	–
*MTGR1*	MC	OS	64.84	2.70–1558.54	0.010	0.86	ab128164	Abcam	1:50
*MUC15*	EC	OS	0.66	0.50–0.87	0.0032	0.79	ab171304	Abcam	1:50
*NSD2*	MC	OS	27.69	1.96–391.91	0.0140	0.86	AMAb90848	Sigma-Aldrich	1:25
*NUP155*	CCC	DSS	27.93	2.98–261.42	0.0035	0.86	ab157104	Abcam	1:100
*PARPBP*	MC	OS	8.079	1.32–49.28	0.0240	0.85	ab211634	Abcam	1:50
*PDE4DIP*	EC	OS	1.00	0.9964–0.9996	0.013	0.75	HPA008162	Sigma-Aldrich	1:25
*PIK3CA*	EC	OS	1.0033	1.001–1.0057	0.0044	0.77	SAB2701957	Sigma-Aldrich	1:100
*RIMBP2*	EC	DSS	0.59	0.36–0.97	0.0370	0.84	ab128045	Abcam	1:25
*RPL13A*	CCC	OS	4.15	1.83–9.39	0.0006	0.79	ab209829	Abcam	1:25
*RPL37*	CCC	OS	4.95	1.91–12.84	0.0010	0.80	SAB4502669	Sigma-Aldrich	1:100
*SETD3*	CCC	OS	79.39	6.063–1039.64	0.0009	0.81	HPA003591	Sigma-Aldrich	1:25
*SMYD2*	CCC	DSS	56.79	3.92–822.37	0.0031	0.89	PA5-51339	ThermoFisher	1:100
*TRIO*	CCC	OS	9.40	2.60–34.03	0.0006	0.83	HPA008157	Sigma-Aldrich	1:25
*ZDHHC2*	MC	OS	0.34	0.15–0.78	0.011	0.84	ab174967	Abcam	1:50

*All members of the study cohort had P-values below 0.05 and concordance index (C-index) values greater than or equal to 0.75. An optimized dilution factor could be determined for all proteins except KNTC1. Monoclonal antibodies were used for CCT5, CENPI, NSD2, NUP155, and RIMBP2. The remaining antibodies were polyclonal antibodies. The statistics data [hazard ratio (HR), 95% confidence interval (CI), P-value, C-index] refers to the Cox regression analysis with raw RNA sequencing (RNA-seq) read counts. Antibody, company and optimized dilution refers to the IHC analysis*.

### Immunohistochemical Analysis and Evaluation

The Human Protein Atlas (HPA) was used as a primary source for the selection of suitable antibodies for each protein (Table 2) (9, 10). Fifteen tumor samples representing different histotypes (CCC, EC, HGSC, MC) and International Federation of Gynecology and Obstetrics (FIGO) stages were used to optimize the selected antibodies. In brief, each antibody was optimized using full-face FFPE sections from 2/15 samples corresponding to the histotype to be tested. Thereafter, a positive control was chosen by testing the optimal antibody dilution on a TMA containing the 15 samples in the optimization panel.

Four micrometer TMA sections for the patient cohort were pretreated using the Dako PTLink system (pH 9) and immunostained with respective antibodies (Table 2) using the Dako Autostainer Plus (Agilent Technologies). Liquid DAB (3,3′-diaminobenzidine) was used as chromogen and EnVision FLEX hematoxylin (Link) as counterstain. Finally, the sections were rinsed using deionized water, dehydrated in ethanol (70, 95, and 100% ethanol), cleared in xylene and mounted. The immunostained sections were scanned using the ZEISS Axio Scan.Z1 and visualized using ZEN lite software (Carl Zeiss Microscopy) to enable easier evaluation of the immunostained TMA cores. A board certified pathologist (AK), blinded to patient survival outcome, performed the immunohistochemical evaluation. An immunoreactive score (H-score) was determined for each tumor core based on percentages of stained tumor cells and staining intensities (weak = 1, moderate = 2, strong = 3) (11). The resulting H-score was based on the mean of the triplicate cores. The staining intensities in normal cells and tumor stroma cells were also determined, wherein positively stained stroma was evaluated in fibroblasts and not in tumor infiltrating lymphocytes. Homogenous staining herein defines IHC samples having either weak, moderate or strong protein staining across one sample triplicate, whereas non-homogenous protein staining is defined as a mix of the staining intensities (i.e., weak-moderate, moderate-strong, weak-moderate-strong) in at least 2/3 TMA cores.

### Statistical Analysis

The statistical analyses were conducted in R/Bioconductor (v. 3.6.0) with *P*-values < 0.05 (two-sided) for statistical significance. To generate histotype-specific prognostic signatures, univariable Cox proportional hazard models were used to correlate RNA-seq expression data with survival outcome (OS/DSS) in respective histotypes (CCC, EC, and MC). *P*-values for determining possible confounding factors between clinicopathological parameters and hisototype as well as positive/negative protein expression were calculated using two-tailed Fisher's exact test (tableone v. 0.10.0) (12). Kaplan-Meier plots in X-tile software (v. 3.6.1) were used to dichotomize H-score cutoff values into positive and negative protein expression groups (13). Kaplan-Meier survival analyses for determining the clinical relevance of protein expression (H-score values) with survival data (OS/DSS) were performed using R packages survival (v. 2.38) and survminer (v. 0.4.4) (14, 15). Univariable and multivariable Cox proportional hazard models were used to evaluate the individual and combined predictive strength (C-index) of the CCC-, EC-, and MC-associated biomarkers. Moreover, multivariable Cox proportional hazard models were also utilized to determine predictive models for the biomarkers related to CCC, EC, and MC in combination with established clinical parameters (age, stage, CA125, ploidy). External validation of the biomarkers' clinical relevance was performed using the Kaplan-Meier (KM) plotter online tool (https://kmplot.com/analysis/) for overall survival of ovarian carcinoma patients (*n* = 1,657) with Affymetrix gene expression microarray data (16). The association with event probability, i.e., increased or decreased survival risk, was also assessed with forest plots (forestplot v. 1.9) (17). The relationship between RNA expression log2 values (raw RNA-seq read counts) and protein expression (H-score values) was compared using ggplot2 (v. 3.1.0) and the statistical difference was evaluated using Wilcoxon test (18). The study cohort was validated in line with the REMARK reporting recommendations for prognostic biomarkers (Supplementary Table 1) (19).

## Results

### Selection of Candidate Genes Associated With Ovarian Carcinoma Prognosis in Different Histotypes

To identify genes associated with prognosis and specific histotypes (CCC, EC and MC), univariable Cox proportional hazards models were calculated using raw RNA-seq read counts and survival outcome (OS, DSS). In total, 3,557 (OS) and 1,827 (DSS) genes with *P*-values < 0.05 were identified for CCC, and 1,440 (OS) and 522 (DSS) genes for EC. For MC, 970 genes were significantly linked to OS. A selection of 11 genes associated with CCC (*ARPC2, CCT5, DDX24, GNB1, KCTD10, NUP155, RPL13A, RPL37, SETD3, SMYD2, TRIO*), 8 with EC (*ABCA12, CECR1, ESRRG, KIF26B, MUC15, PDE4DIP, PIK3CA, RIMBP2*), and 10 with MC (*CENPI, CHEK1, FOXM1, KIF15, KIF23, KNTC1, MTGR1, NSD2, PARPBP, ZDHHC2*) were chosen among the top 50 genes according to the selection criteria with C-index ranging between 0.75 and 0.91 (Table 2). The majority of the 29 selected genes were only significant (*P*-value < 0.05) in the above specified histotypes, with the exception of *KIF23* (DSS, C-index = 0.72), *PDE4DIP* (OS C-index = 0.69, DSS C-index = 0.77), and *ZDHHC2* (OS C-index = 0.67, DSS C-index = 0.76) that were further significant in CCC, as well as *KIF26B* (OS C-index = 0.74) in MC.

### IHC Analysis Revealed Aberrant Protein Expression Patterns

Optimal antibody dilutions were determined for 28/29 biomarkers (Table 2). KNTC1 showed negative protein staining at 1:25 antibody dilution and was therefore excluded from further analysis. IHC analysis was performed for the remaining 28 biomarkers using optimal antibody dilutions on samples corresponding to the histotype to be tested (Table 2). Dichotomization of H-scores using X-tile software could be determined for 17/29 proteins. Positive staining was interpreted as H-score >210 for ARPC2, >150 for CCT5, >73 for CECR1, >17 for CHEK1, >55 for FOXM1, >193 for GNB1, >267 for KCTD10, >180 for KIF23, >40 for KIF26B, >200 for NUP155, >167 for PARPBP, >100 for PIK3CA, >200 for RPL13A, >73 for RPL37, >200 for SETD3, >180 for SMYD2, and >150 for TRIO.

**Figure 1:**
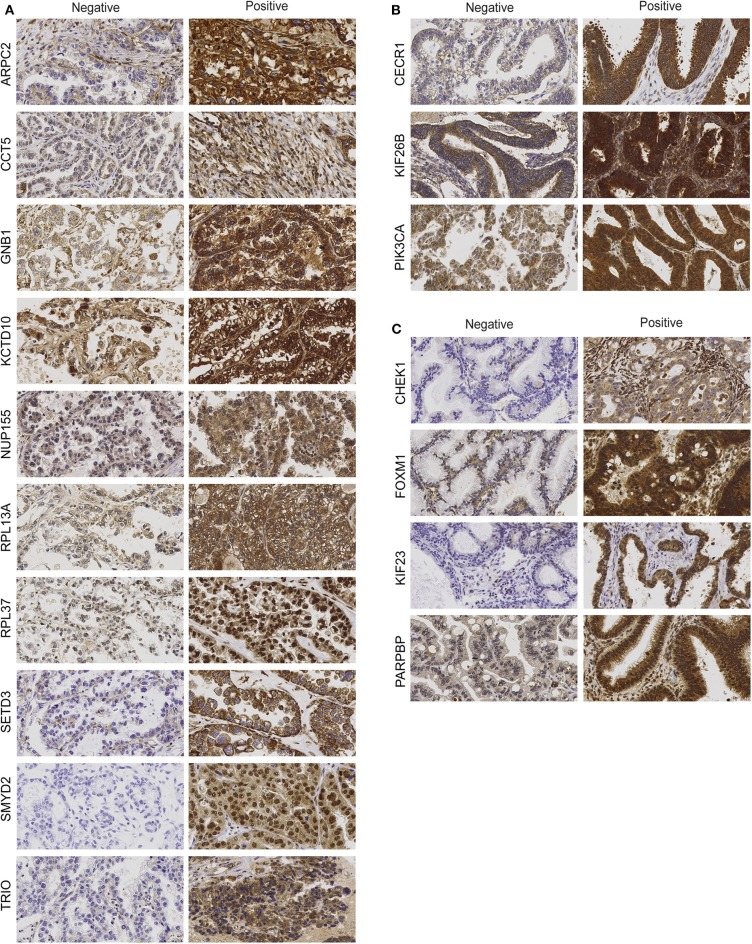
Protein expression of clear-cell (CCC)-associated biomarkers **(A)**, endometrioid (EC)-associated biomarkers **(B)**, and mucinous ovarian carcinoma (MC)-associated biomarkers **(C)**. Representative immunohistochemical staining intensities showing protein expression (negative vs. positive) in ovarian tumor cells for histotype-associated biomarkers (400 × magnification).

The IHC analysis revealed that the CCC-associated biomarkers (ARPC2, CCT5, GNB1, KCTD10, NUP155, RPL13A, RPL37, SETD3, SMYD2, TRIO) were mainly expressed in the cytoplasm of tumor cells (Figure 1A). Apart from cytoplasmic staining, RPL13A, RPL37, and SMYD2 also showed nuclei staining. Positive immunostaining was prevalent for GNB1 (68%), KCTD10 (59%), and RPL37 (97%), whereas negative immunostaining was primarily found for the remaining CCC-associated biomarkers [ARPC2 (24%), CCT5 (49%), NUP155 (5%), RPL13A (3%), SETD3 (16%), SMYD2 (3%), and TRIO (8%) (percentages of positive immunostaining is shown in parenthesis)]. GNB1 immunostaining patterns (weak, moderate or strong staining) were generally homogenous for all samples. The nine remaining biomarkers were found to have partially non-homogenous immunostaining patterns were (weak-moderate or moderate-strong staining) in at least 2/3 of the TMA cores. Apart from age for ARPC2 and ploidy for SETD3, none of the CCC-associated biomarkers showed any association between protein expression and clinicopathological data (Supplementary Table 2). Protein staining for biomarkers related to EC (CECR1, KIF26B, PIK3CA) was mainly localized to the cytoplasm of tumor cells (Figure 1B). CECR1, KIF26B, and PIK3CA demonstrated mainly positive immunostaining with 76, 98, and 85%, respectively. Further, the EC-related biomarkers revealed partially non-homogenous immunostaining pattern for up to 16/46 samples. Stromal staining (majority of weak staining) was found in 31/46 patient samples for PIK3CA. MC-associated biomarkers (CHEK1, FOXM1, KIF23, PARPBP) were also shown to display cytoplasmic staining (Figure 1C). Positive immunostaining was mainly shown for CHEK1 (83%) and FOXM1 (86%), and predominantly negative immunostaining for KIF23 (7%) and PARPBP (41%). Generally, the staining pattern was homogenous for the MC-associated biomarkers. However, CHEK1 and PARPBP demonstrated partially non-homogenous staining pattern for a few samples. No association was found between EC- or MC-associated biomarker protein expression and clinicopathological data (Supplementary Tables 3, 4).

The relationship between protein expression and RNA expression levels was further examined by comparing both H-score and raw RNA-seq read counts in log2 scale. Significantly higher RNA expression levels (*n* = 17) were found for all biomarkers related to CCC compared to protein expression (*n* = 37) (Supplementary Figure 5). A similar trend was demonstrated for EC-associated biomarkers (PIK3CA and CECR1) with higher RNA expression (*n* = 17) in comparison with protein expression (*n* = 46). KIF26B showed no significant difference between the two expression types. For biomarkers related to MC, no significant difference between protein (*n* = 29) and RNA expression (*n* = 11) was found for CHEK1, FOXM1, and PARPBP. The RNA-protein difference for KIF23 was barely significant (*P*-value = 0.049).

### CCC-Related Biomarkers Improved the Predictive Performance of Prognostic Models

**Figure 2:**
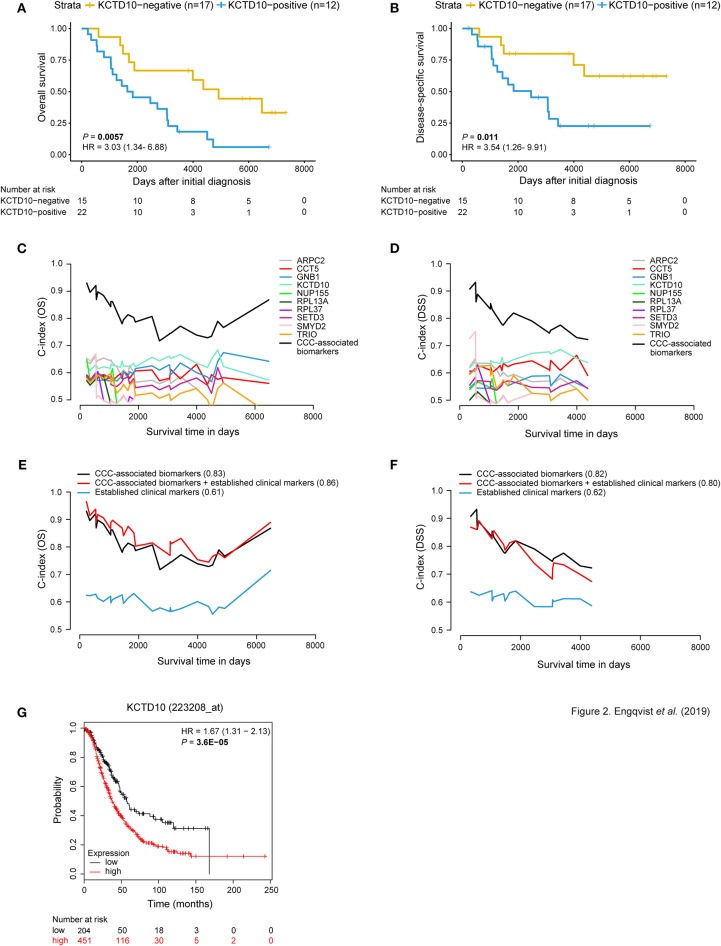
Prognostic value of CCC-associated biomarkers. Kaplan-Meier survival plots **(A,B)** showing patient survival [overall survival (OS)/disease-specific survival (DSS)] in relation to dichotomized KCTD10 protein expression. Patients with KCTD10-positive staining (blue curve) correlated with both shorter OS and DSS [*P*-value = 0.0057, hazard ratio (HR) = 3.03 (95% confidence interval (CI) 1.34–6.88); *P*-value = 0.011, HR = 3.54 (95% CI 1.26–9.91)]. The x-axis depict days after initial diagnosis and the y-axis survival outcome (OS/DSS). The number of patients at risk by time (days after initial diagnosis) is shown below the Kaplan-Meier plot. Univariable and multivariable time-dependent area under the ROC curve [AUC(t)] plots **(C,D)** illustrating the predictive performance of each model over time and a significantly improved predictive model when combining the individual CCC-associated biomarkers (black curve) for OS and DSS. Multivariable survival plots for OS and DSS showing improved outcome prediction for CCC-associated biomarkers in comparison with established clinical markers **(E,F)**. The outcome prediction concordance index (C-index) values are shown in parentheses. Further, the addition of protein expression status of CCC-associated biomarkers to established clinical markers resulted in improved outcome prediction (C-index = 0.86) for OS. Survival analysis was adjusted for age, stage, CA125, ploidy. The x-axis depicts survival time in days and the y-axis C-index values (OS/DSS). The prognostic value of each CCC-associated biomarker was validated using KM plotter for OS in HGSC and EC histotypes (*n* = 655) **(G)**. Here, the prognostic value of *KCTD10* is validated wherein patients with *KCTD10*-positive gene expression (patient samples with expression levels above the median) is shown in red and *KCTD10*-negative gene expression (patient samples with expression levels below the median) is shown in black. The number of patients at risk is indicated below the Kaplan-Meier plot. Cox proportional hazard models and log-rank tests were used to calculate HR, 95% confidence interval, and log rank *P*-value for Kaplan-Meier survival analysis and KM Plotter validation analysis.

Protein expression for CCC-associated biomarkers (10/29 biomarkers) was significantly associated with survival outcome (OS and/or DSS) using Kaplan-Meier survival analysis and log-rank tests (*P*-values < 0.05) with dichotomized protein expression according to H-score cutoffs (Figures 2A,B, Supplementary Figure 1). GNB1, NUP155, RPL13A, and SETD3 protein expression revealed a significant association with OS. Moreover, patients with positive protein expression for ARPC2, KCTD10, SMYD2, and TRIO demonstrated significantly shorter OS and DSS. CCT5- and RPL37-negativity were associated with shorter DSS and both shorter OS and DSS, respectively. These findings were in agreement with the association between RNA expression and clinical outcome, with the exception for CCT5 and RPL37, wherein positive gene expression of these proteins correlated with shorter DSS and OS, respectively. A comparison between RNA and protein expression demonstrated consistently higher RNA expression levels than protein expression levels for all CCC-associated biomarkers (Supplementary Figure 5A). Moreover, CCT5 and RPL37 were shown to be associated with decreased risk of mortality (hazard ratio (HR) values below 1), whereas the remaining CCC-associated biomarkers were associated with increased risk of mortality (HR values above 1) (Supplementary Figure 4).

Interestingly, univariable models containing the CCC-associated biomarkers revealed predictive potential for OS and DSS. In addition, predictive models containing all 10 CCC-associated biomarkers (OS C-index = 0.83, DSS C-index = 0.82) outperformed (increased C-index) models for individual markers and established clinical markers (age, CA125, ploidy, and stage) (Figures 2C–F). KCDT10 showed the highest individual prognostic potential (OS C-index = 0.63, DSS C-index = 0.65). Moreover, multivariable survival analysis demonstrated an improved predictive performance for OS when combining the CCC-associated biomarkers with the established markers from C-index 0.61–0.86 (Figures 2E,F, Supplementary Table 5). However, the C-index (0.80) for the combined DSS model (CCC-associated biomarkers and established clinical markers) was lower than for the CCC-associated biomarkers alone. Including PITHD1 protein expression status to the OS and DSS models resulted in a further improvement of the C-indices [C-index = 0.88 (OS), 0.89 (DSS)] (7).

The prognostic potential of the CCC-related biomarkers was validated in an external gene expression dataset (KM plotter) for OS of ovarian cancer patients (*n* = 655 for CCT5, KCTD10, and SETD3; *n* = 1,656 for the remaining CCC-related biomarkers). The Kaplan-Meier survival plots were dichotomized according to expression levels above the median (i.e., positive expression) and below the median (i.e., negative expression) (Figure 2G, Supplementary Figure 6, Supplementary Table 8). *CCT5, GNB1, KCTD10, NUP155*, and *SETD3* gene expression was significantly correlated with shorter OS (*P*-value < 0.05). *ARPC2* and *RPL37* gene expression showed a tendency to shorter OS, but were not statistically significant. Lastly, negative gene expression of *RPL13A, SMYD2*, and *TRIO* was significantly correlated with shorter OS.

### EC-Associated Biomarkers Demonstrated Prognostic Value and Improved Predictive Performance

**Figure 3:**
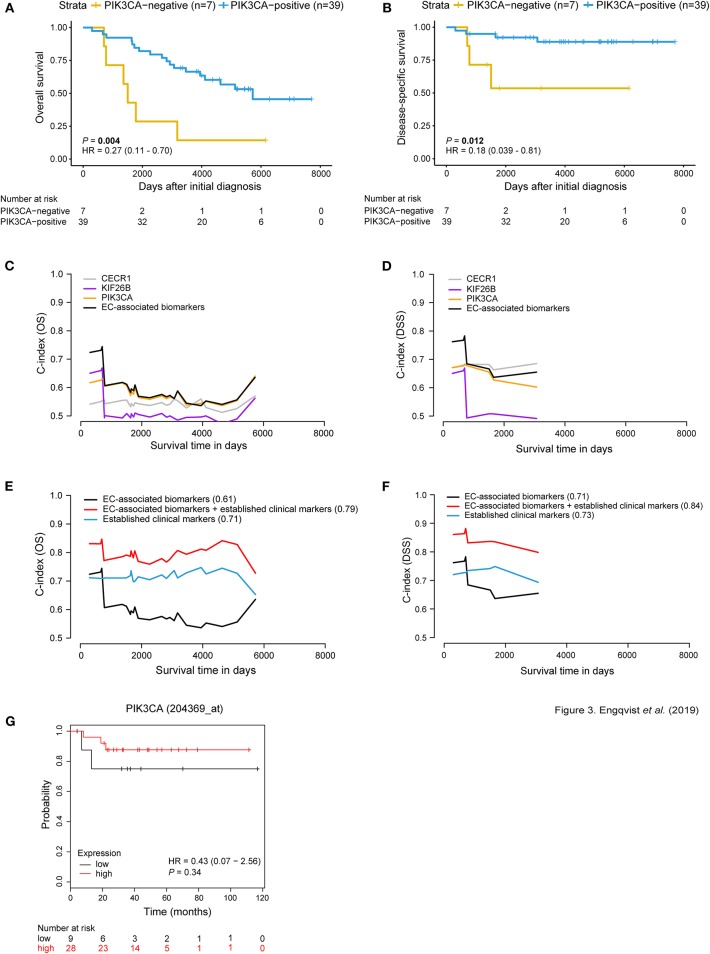
Prognostic significance of EC-related biomarkers. Survival analysis using Kaplan-Meier plots **(A,B)** depicting OS and DSS patient survival in relation to dichotomized PIK3CA protein expression. Positive PIK3CA protein expression (blue curve) was associated with longer OS and DSS [*P*-value = 0.004, HR = 0.27 (95% CI 0.11–0.70); *P*-value = 0.012, HR = 0.18 (95% CI 0.039–0.81)]. The x-axis depicts days after initial diagnosis and the y-axis survival outcome (OS/DSS). The patient numbers at risk by time (days after initial diagnosis) is shown below the Kaplan-Meier plot. Univariable and multivariable survival analysis for OS/DSS **(C,D)** showing AUC(t) plots of individual EC-associated biomarkers and in combination (black curve). Multivariable survival analysis **(E,F)** illustrating improved predictive performance for both OS and DSS when combining protein expression of the EC-related biomarkers with established clinical markers (C-index = 0.79; 0.84). C-index values for each curve are shown in parentheses. Survival analysis was adjusted for age, stage, CA125, ploidy. The x-axis depicts survival time in days and the y-axis C-index values (OS/DSS). KM plotter was used to test the prognostic value of the EC-associated biomarkers in an external ovarian carcinoma dataset containing EC patients (*n* = 37). Here, *PIK3CA* gene expression is not associated with OS **(G)**. However, RNA and protein expression for PIK3CA displayed similar trends, where patient samples with low expression shorter OS. Cox proportional hazard model and log-rank tests were used to calculate HR, 95% confidence interval, and log rank *P*-value for Kaplan-Meier survival analysis and KM Plotter validation analysis.

Kaplan-Meier survival analysis (dichotomized according to H-score cut offs) revealed an association between CECR1-negativity and shorter DSS, and negative KIF26B and PIK3CA protein expression and both shorter OS and DSS (Figures 3A,B, Supplementary Figure 2). These results are in line with the RNA-seq results (for 17 RNA-seq samples) for *CECR1* and *KIF26B* genes wherein higher RNA expression was found in long-term survivors. Contradictive to protein expression, *PIK3CA* showed lower RNA expression in relation to long-term survival. Similar to the protein-RNA comparison for the CCC-associated biomarkers, CECR1 and PIK3CA gene expression levels were elevated compared to protein expression levels. No significant difference in protein and RNA expression was found for KIF26B (Supplementary Figure 5B). Patients with negative CECR1 (DSS), KIF26B (OS, DSS) or RPL37 (OS, DSS) protein expression had a decreased risk of mortality (Supplementary Figure 4).

Univariable analysis of the EC-associated proteins showed predictive performance, wherein PIK3CA showed the highest C-index of 0.59 for OS, and CECR1 with C-index of 0.68 for DSS (Figures 3C,D, Supplementary Table 6). An improved predictive performance was further demonstrated when combining the EC-associated proteins (OS C-index = 0.61, DSS C-index = 0.71). Further, a multivariable predictive model containing EC-associated proteins and established clinical markers (age, stage, CA125, ploidy) revealed an overall improved predictive performance (OS C-index = 0.79, DSS C-index = 0.84) (Figures 3E,F). Furthermore, survival analysis for the EC-related biomarkers was performed in an external gene expression dataset containing EC patients. No significant difference between gene expression and OS was shown for the EC-associated biomarkers using KM plotter for EC patients (*n* = 37). However, the same tendency of correlation between negative gene expression and shorter OS was shown for *KIF26B* and *PIK3CA* in view of their protein expression patterns (Figure 3G, Supplementary Figure 7, Supplementary Table 8).

### MC-Related Biomarkers Improved the Predictive Power of Prognostic Models

**Figure 4:**
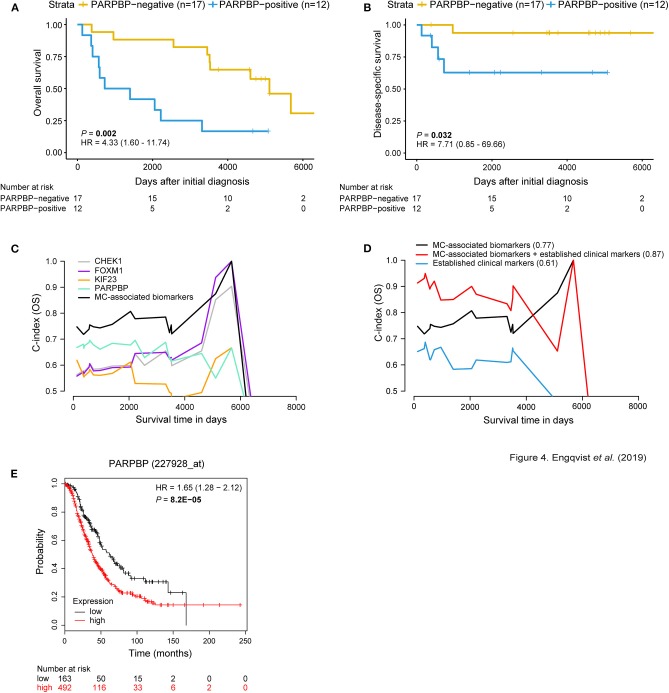
MC-associated biomarkers related to prognosis. Kaplan-Meier survival plots for OS/DSS illustrating dichotomized PARPBP protein expression. Positive PARPBP protein expression correlated with shorter OS and DSS [*P*-value = 0.002, HR = 4.33 (95% CI 1.60–11.74); *P*-value = 0.032, HR = 7.71 (95% CI 0.85–69.66)]. The x-axis depict days after initial diagnosis and the y-axis survival outcome (OS/DSS). The number of patients at risk by time (days after initial diagnosis) is shown below the Kaplan-Meier plot **(A,B)**. Univariable and multivariable survival plots depicting predictive potential [AUC(t) plots] of individual MC-associated biomarkers and improved predictive power when in combination (black curve) for OS **(C)**. Multivariable survival analysis showing improved predictive performance when adding protein expression status of the MC-associated biomarkers to the established clinical markers (age, stage, CA125, ploidy) for OS (C-index = 0.87) **(D)**. C-index values for respective curve is shown in parentheses. The x-axis depict survival time in days and the y-axis C-index values (OS/DSS). External validation using KM plotter for ovarian carcinoma patients was used to test the prognostic value of the biomarkers associated with MC. Here, the prognostic value of PARPBP is validated wherein PARPBP-positive gene expression (red curve) corresponds with shorter OS (*n* = 655 HGSC and EC patients) **(E)**. The number of patients at risk by (days after initial diagnosis) is indicated below the Kaplan-Meier plot. Cox proportional hazard models and log-rank tests were used to calculate HR, 95% confidence interval, and log rank *P*-value.

Survival analyses using Kaplan-Meier curves, dichotomized according to protein expression, and log rank tests (*P*-value < 0.05) revealed prognostic value for the MC-related biomarkers. More specifically, a correlation was found between positive expression of CHEK1, FOXM1, KIF23, and PARPBP and shorter OS. Further, PARPBP-positivity also correlated with shorter DSS (Figures 4A,B, Supplementary Figure 3). This pattern was also shown for the RNA-seq samples (11/29 patients), i.e., positive gene expression corresponded with shorter survival. No significant difference was found between protein expression and gene expression for CHEK1, FOXM1, and PARPBP, whereas the protein-RNA comparison was barely significant for KIF23 (*P*-value = 0.049) (Supplementary Figure 5C). The proteins were also visualized in terms of risk vs. survival, wherein CHEK1- (OS), KIF23- (OS) and PARPBP-positivity (OS, DSS) demonstrated an increased risk of mortality (Supplementary Figure 4). FOXM1 was not included in the forest plot analysis, since none of the four patients in the FOXM1-negative expression group were deceased.

Univariable analysis showed predictive potential for the individual MC-associated biomarkers, with PARPBP having the highest predictive performance (C-index = 0.65) (Supplementary Table 7). An overall improved predictive potential was found when combining the individual MC-associated biomarker potentials (C-index = 0.77). Moreover, a model containing the MC-associated biomarkers and established clinical markers (age, stage, CA125, ploidy) resulted in improved predictive power (C-index = 0.87) (Figures 4C,D). Further, an increased predictive power was demonstrated (C-index = 0.91) when including the protein expression status for GPR158 (linked to MC-associated prognosis) from our previous study to this model (7). KM-plotter confirmed the prognostic value of *CHEK1, KIF23*, and *PARPBP* on the RNA level, wherein high gene expression levels correlated with shorter OS. For *FOXM1*, borderline significance (*P*-value = 0.05) was found between positive gene expression and shorter OS (Figure 4E, Supplementary Figure 8, Supplementary Table 8).

## Discussion

In the current study, 29 genes associated with ovarian cancer histotype-specific (CCC, EC, MC) prognosis were evaluated on the protein expression level using IHC to identify biomarkers for survival. Our findings revealed that the expression levels of 17/29 proteins (10 biomarkers for CCC, three biomarkers for EC, four biomarkers for MC) had a significant impact (positive or negative) on survival in respective histotypes. We performed our analysis using the current ovarian carcinoma histotype classification demonstrating the importance of histotype on origin, clinical and molecular behavior, and prognosis (1, 20). This study performed better in comparison with our previous study in terms of (1) the number of optimized antibodies (28/29 vs. 12/29) and (2) the number of identified biomarkers with prognostic significance on the protein level (17/29 vs. 3/29) (7). This is primarily due to the selection of candidate genes with an overall higher expression level for respective histotypes that could be detected using IHC. Candidate genes associated with the HGSC histotype were not included here since their prognostic potential was relatively low (C-index OS <0.66, C-index DSS <0.69), which may be explained by the heterogeneous nature of Cancer Genome Atlas Research Network (21). In general, the CCC- and EC-associated biomarkers demonstrated lower protein expression compared to observed RNA expression levels (with the exception of no protein/RNA difference for KIF26B). The difference in expression levels may be explained by the use of different detection methods, i.e., protein expression was detected in tumor cells only while transcriptomic expression was determined for the entire tumor mass that contained various cell types. Supporting the importance of histotype-based prognostication, the validated biomarkers (17/29) were only significantly correlated with prognosis in the identified histotypes (for the RNA-seq samples), with the exception of *KIF23*, which was also statistically significant in CCC, and *KIF26B* in MC.

Interestingly, several of the CCC-associated biomarkers (CCT5, NUP155, RPL37, SETD3, SMYD2) have been previously reported to be associated with the p53 tumor suppressor pathway in various types of cancers. p53 can determine the fate of a cell by activating pathways such as growth arrest, cellular senescence or apoptosis. Further, p53 mutations are detected in more than 50% of all human cancers and 25% of tumors lacking p53 mutations have other p53 pathway abnormalities (22). More specifically, *CCT5* mRNA expression was upregulated in p53-mutated breast cancers, and has been reported to play an important role in protein folding, wherein the accumulation of misfolded proteins is associated with various diseases including cancer (23, 24). Nup155, important in nuclear envelope formation, was shown to control mRNA translation of cyclin-dependent kinase inhibitor p21, a key mediator of p53-dependent cell cycle arrest, in murine liver cancer (23, 25, 26). In the normal cell state, p53 protein expression levels are low, and MDM2 and MDMX are important negative regulators of its activity (22, 27). The ribosomal protein RPL37 has been shown to activate p53 in response to genotoxic stress by e.g., binding to and inhibit degradation of Mdm2 and p53, downregulate MdmX protein levels, and upregulate p21 (28). The actin histidine methyltransferase SETD3 upregulated p53-dependent activation of apoptosis in response to doxorubicin treatment in colon cancer cells (29, 30). Lastly, the lysine methyltransferase SMYD2 has been shown to repress the tumor suppressive function of p53 via DNA methylation (31). A recent study demonstrated worse prognosis for p53 negative/overexpressed tumors in comparison with p53 positive tumors in a cohort of CCC, EC and endometrial cancers (*n* = 97) (3). Herein, survival analysis using Kaplan-Meier plots revealed significant correlations between positive expression of SETD3 and SMYD2 and worse clinical outcomes (OS and DSS) which is in line with our RNA-seq results. SETD3-positivity has been reported to be associated with poor prognosis in hepatocellular carcinoma (32). Similar to our expression profile, high levels of SMYD2 expression has been associated with unfavorable prognosis in different cancer types, such as breast cancer (mRNA expression) and cervical cancer (protein expression) (33, 34). A recent study reported overexpression of SMYD2 in cancer vs. normal tissue, and associated higher expression of SMYD2 with enhanced proliferation in HGSC (35). NUP155-positivity correlated with shorter OS in CCC, while esophageal squamous cell carcinoma showed an association between low expression of NUP155 with shorter OS (36). Contradictive to the RNA-seq results, CCT5- and RPL37-positivity correlated with longer DSS, and OS and DSS, respectively. No previous association for CCT5 and RPL37 expression with survival outcome has been shown.

Kaplan-Meier survival analysis for the remaining CCC-associated biomarkers showed a significant correlation between positive expression of ARPC2, GNB1, KCTD10, RPL13A, and TRIO and unfavorable prognosis (OS or OS/DSS), which is in agreement with the RNA-seq results. ARPC2 plays a crucial role in actin polymerization and elevated expression thereof was correlated with unfavorable outcome for breast cancer patients (37). Moreover, a potential drug, benproperine, has been suggested to target ARPC2, and thus inhibit cell migration and metastasis in cell and mouse studies (38). In colon cancer, positive expression of GNB1, which is part of the RAS-BRAF-MAPK-ERK pathway, was associated with longer OS (39, 40). KCTD10 plays a role in DNA repair, DNA replication and cell-cycle control, and has been identified as a key gene in pancreatic carcinogenesis (41). Little could be found linking RPL13A to prognosis in cancer (42). Interestingly, RPL13A has been reported to be stable independent of disease progression in ovarian cancer patients (*n* = 50; 25 normal, benign or borderline patient samples, 25 malignant epithelial tumors), and a suitable reference gene for qPCR (43). It should however be noted that only 4/25 cancer samples were characterized as CCC. TRIO plays a role in cell proliferation and progression of cancer, wherein higher protein expression has been shown to be associated with worse outcome (OS) (44). To the best of our knowledge, we are the first to report an association between CCC-related biomarkers (ARPC2, CCT5, GNB1, KCTD10, NUP155, RPL13A, RPL37, SETD3, SMYD2, TRIO) and prognosis. Additionally, the majority (8/10) of CCC-associated biomarkers could be validated in the KM plotter external cohort, consisting of geographically different populations. However, *RPL13A, SMYD2*, and *TRIO* showed opposing prognostic significance, i.e., positive gene expression correlated with longer survival. This could be explained by the fact that the majority of KM plotter ovarian carcinoma samples were comprised of HGSC (*n* = 1,232) and a few EC patients (*n* = 62). Unfortunately, there are no public databases comprising expression data for CCC or MC patients.

The protein expression levels of the EC-associated biomarkers (CECR1-negativity and KIF26B-negativity) were significantly correlated with unfavorable survival outcomes (DSS; OS and DSS), which was in line with the RNA-seq data. Shorter OS and DSS were correlated with PIK3CA-negativity on the protein expression level, but *PIK3CA*-positivity on the RNA level. However, the KM plotter data and protein expression data showed a similar association (however *P*-value > 0.05) with survival outcome. No connection with prognosis in ovarian carcinoma has previously been shown for CECR1. In glioblastoma, upregulated CECR1 has however been shown to contribute to tumor expansion and angiogenesis (45). Upregulation of KIF26B increased proliferation and migration in ovarian cancer cell lines (46). Although not statistically significant in KM plotter (*P*-value = 0.076), KIF26B-positivity correlated with favorable outcomes in EC patients using RNA-seq and IHC analysis. Interestingly, *KIF26B*-positivity was associated with unfavorable OS using HGSC data from TCGA-OV and KM plotter (46). This also highlights the importance of histotype-based survival analyses. PIK3CA is frequently mutated in ovarian EC (47). Few studies have evaluated PIK3CA in view of prognosis for ovarian EC. One study coupled mutations in Pik3ca or Trp53 with shorter survival and metastasis in an EC mouse model (48). As far as we know, we are the first to report a connection between CECR1, KIF26B, and PIK3CA protein expression and prognosis in ovarian EC patients.

Survival analysis showed that the RNA expression levels for 4/10 MC-related biomarkers (*CHEK1, FOXM1, KIF23, PARPBP*) significantly correlated with their respective protein expression levels, as well as, the association of positive expression with shorter OS. CHEK1, involved in checkpoint mediated cell cycle arrest in response to e.g., DNA damage, has been reported to act as BRCA-like tumor suppressors when mutated in hereditary ovarian cancer (49). In breast cancer, *CHEK1* mRNA expression and phosphorylated CHEK1 protein have demonstrated prognostic value in breast cancer-related death (50). Mutations in TP53 have been suggested to contribute to FOXM1 overexpression, and the FOXM1 transcription factor network to be altered in the majority (87%) of (21). Further, FOXM1 plays a role in cell proliferation and has been linked to epithelial ovarian carcinoma prognosis wherein FOXM1-positivity was associated with shorter OS (51). KIF23 has been suggested to promote cell proliferation and migration, and KIF23-expression to be coupled to poor OS prognosis in ovarian tumors (52). PARPBP has been demonstrated to be a negative regulator of homologous recombination and to be involved in cell cycle regulation and contribute to unfavorable outcomes in hepatocellular carcinoma (53, 54). Moreover, PARPBP has been suggested to be activated by FOXM1 in gastric cancer cells (55). Our prognostic results were further in line with the KM plotter data (HGSC and EC patients) [with the exception of borderline significance (*P*-value = 0.05) for FOXM1]. To the best of our knowledge, no association between CHEK1, FOXM1, KIF23, or PARRPBP expression and prognosis has been reported for mucinous ovarian carcinoma. Furthermore, combined predictive models comprising protein expression status of all validated biomarkers related to CCC, EC, and MC together with established clinical markers improved the predictive power (increased C-index values) compared with models containing only established clinical markers, further strengthening the importance of these biomarkers. Interestingly, the addition of protein expression status of our previously identified PITHD1 (CCC) and GPR158 (MC) biomarkers to the predictive models (CCC- or MC-associated biomarkers and established clinical markers) further increased the predictive power [C-index = 0.88 (OS), 0.89 (DSS), and C-index = 0.91 (OS)].

Our study has many strengths. It involves the validation of prognosis-related biomarkers within 3/5 of the major histotypes in early-stage ovarian carcinoma. To date, such information of histotype-specific prognostic biomarkers is limited for CCC, EC, and MC, particularly for early-stage disease. IHC (a standard method of testing protein expression) was used in the present study, enabling easy detection of the identified biomarkers in the clinic. The biggest drawback of the present study is the small sample size (*n* = 112). Stratification of the cohort by histotype and survival led to relatively small groups, e.g., three CCC-related biomarkers (RPL13A, RPL37, SMYD2) only contained one patient, resulting in questionable statistics. This is however a general problem for studies on ovarian carcinomas since it is a rare disease (541 patients diagnosed in 2016 in Sweden) (56). Further, the majority of epithelial ovarian carcinoma patients are diagnosed at late stages (stage III+IV: 62%) vs. early stages (stage I+II: 36%) (57). Lastly, it has been reported that the highest incidence of early-stage ovarian carcinomas are classified as HGSC and not the studied histotypes [HGSC (35.5%), LGSC (1.9%), EC (26.6%), MC (7.5%), CCC (26.2%)] (58). In the IHC analysis, we were able to extend our RNA-sequenced patient group (*n* = 45) with additional FFPE samples. However, larger clinical studies involving larger patient cohorts are needed, using patients from different regions and/or countries, to further validate our conclusions and reduce the number of histotype-specific biomarkers thereby enabling practical clinical application of each panel. A further limitation is the difficulty to validate our results in external cohorts within corresponding histotypes, since most cohorts are primarily comprised of HGSC patients (e.g., the TCGA ovarian carcinoma cohort and KM plotter). Only 3/17 biomarkers (CCT5, RPL37, PIK3CA) showed opposing protein expression vs. RNA expression in relation to clinical outcome. The discrepancy may be explained by e.g., the different techniques used (transcriptomic expression of all cell types vs. protein expression in tumor cells only), or regulation of gene expression (59). In summary, we validated 17 novel histotype-specific prognostic biomarkers; 10 biomarkers for CCC, three biomarkers for EC and four biomarkers for MC, that to the best of our knowledge have not previously been connected with ovarian CCC-, EC-, or MC-prognosis in early-stage ovarian carcinoma. The validated proteins may better predict the risk (high or low) of CCC-, EC-, or MC-associated survival and may thereby be used as potential targets to guide clinical therapy decisions.

## Data Availability Statement

The datasets analyzed in this study can be found in the NCBI Gene Expression Omnibus (http://www.ncbi.nlm.nih.gov/geo/) (GSE40744).

## Ethics Statement

The studies involving human participants were reviewed and approved by the Regional Ethical Review Board (case number 767-14, Gothenburg, Sweden). The ethical review board further approved a waiver of written consent to use the tumor specimens.

## Author Contributions

KH and PK were responsible for overall study concept, design of experiments, and collection of clinical data. HE and TP performed the statistical analyses. AK and ER performed the pathological reclassification of the histotypes, and AK the evaluation of stained tissue sections. KS provided technical support relating to ovarian carcinoma. HE planned, prepared and performed the IHC experiments, analyzed the data, and wrote the manuscript. All authors reviewed, edited, and approved the final manuscript.

### Conflict of Interest

The authors declare that the research was conducted in the absence of any commercial or financial relationships that could be construed as a potential conflict of interest.
