# Supplementary material for: Validation of Novel Prognostic Biomarkers for Early-Stage Clear-Cell, Endometrioid and Mucinous Ovarian Carcinomas Using Immunohistochemistry
Source: Front Oncol. 2020 Feb 18;10:162. doi: 10.3389/fonc.2020.00162 (PMC7040170; doi:10.3389/fonc.2020.00162)
Supplement: Supplementary file 1 [file Data_Sheet_1.PDF]

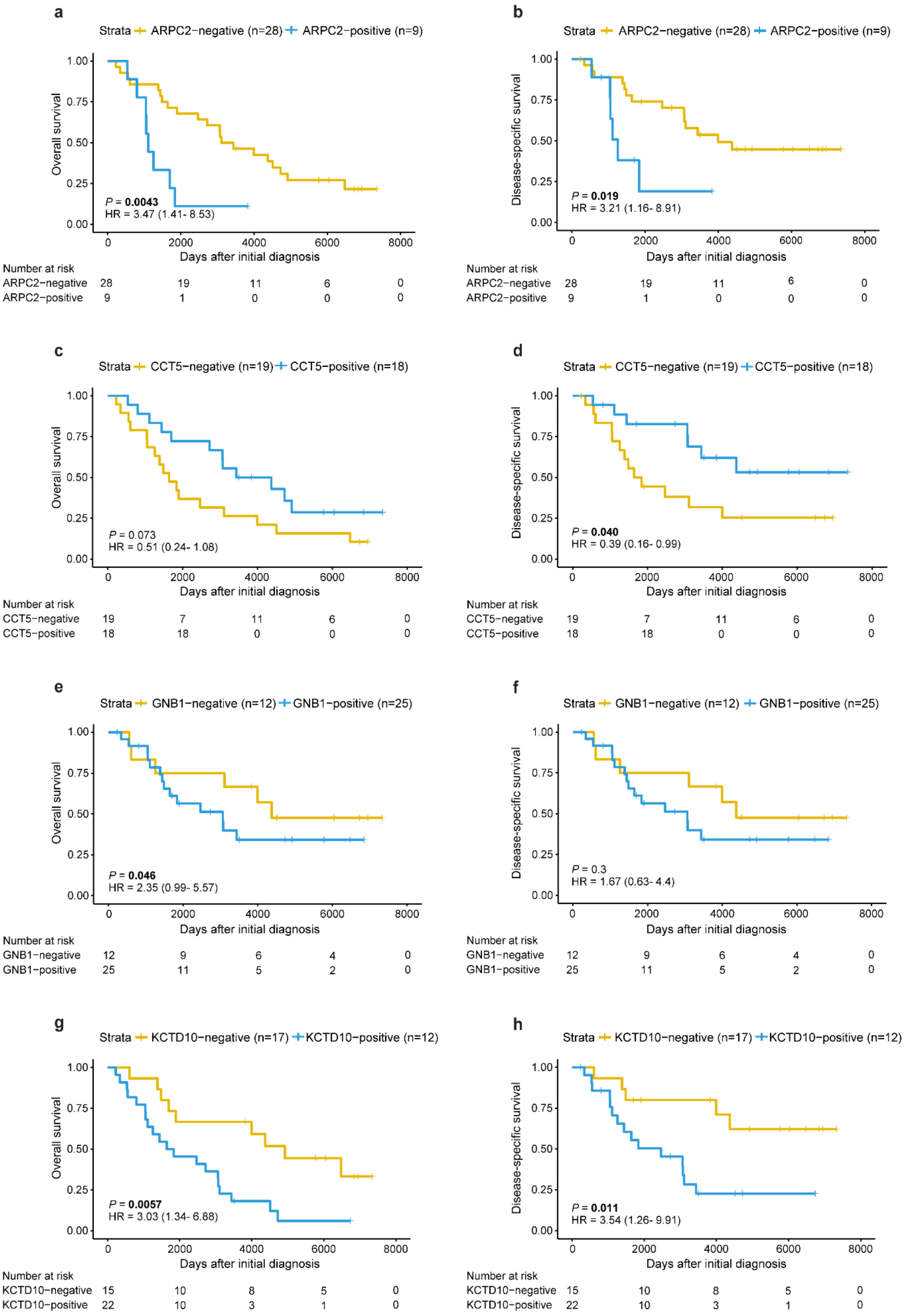

Supplementary Figure 1. Engqvist *et al.* (2019)

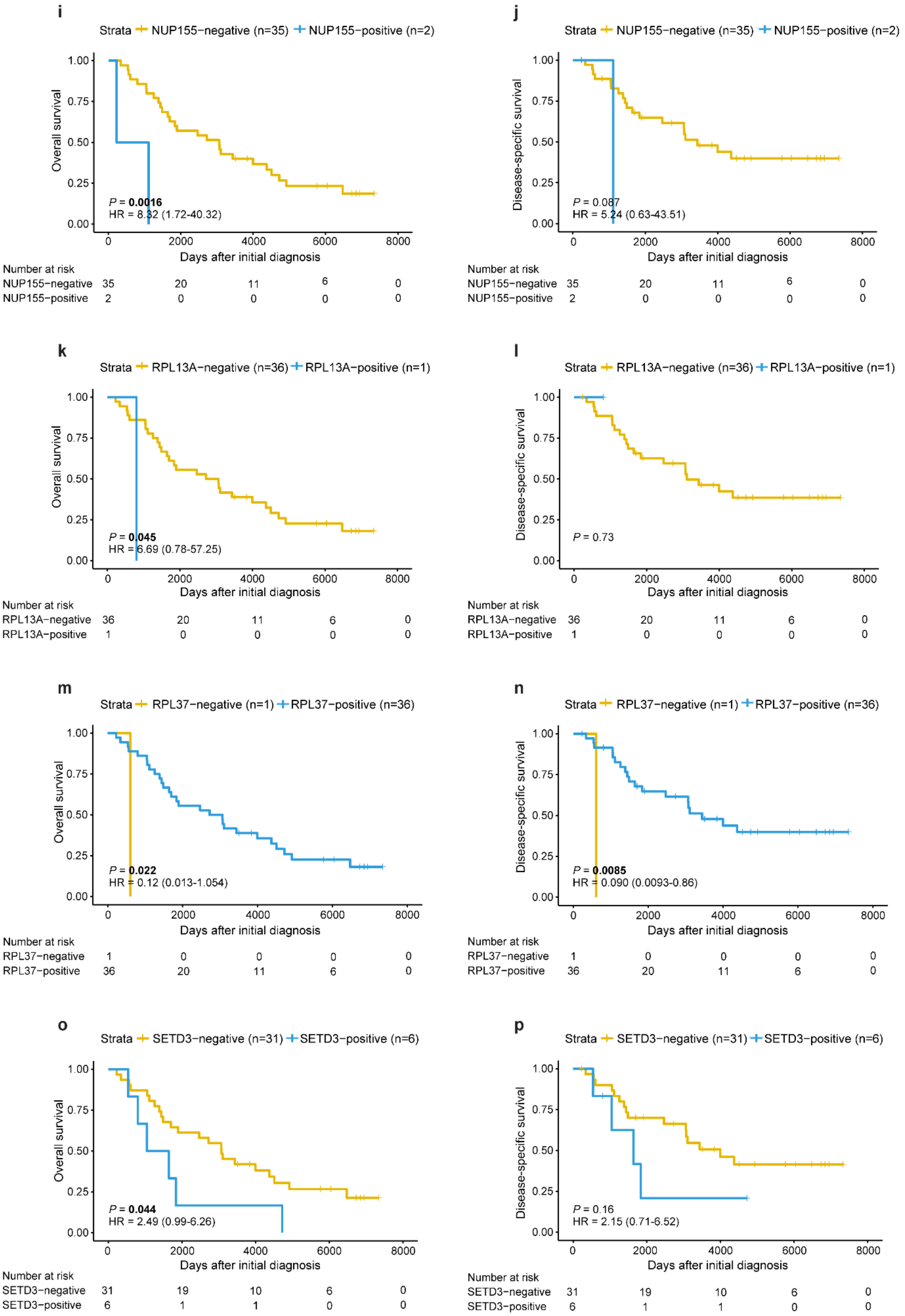

Supplementary Figure 1. Engqvist *et al.* (2019)

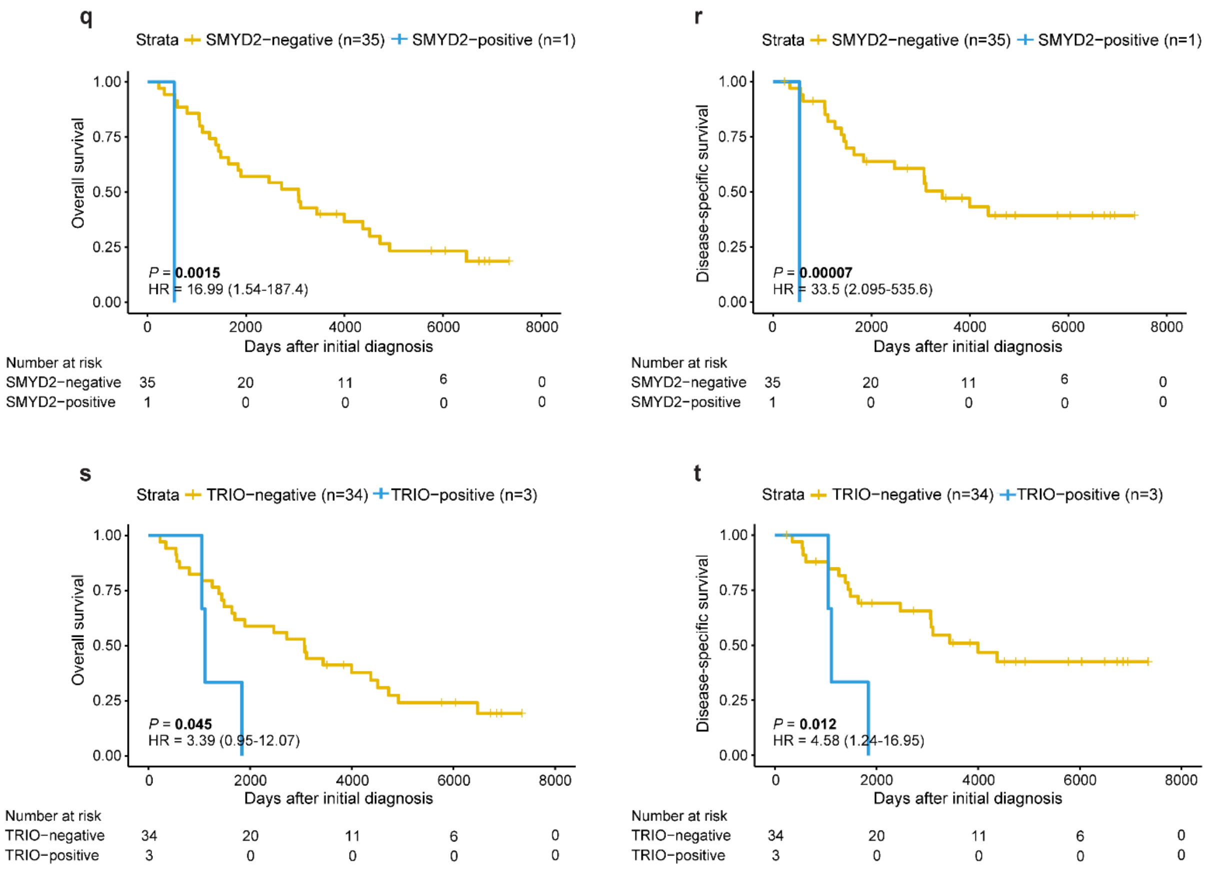

Supplementary Figure 1. Engqvist *et al.* (2019)

Supplementary Figure 1: Survival analysis for CCC-associated biomarkers. Kaplan-Meier survival analysis showing the relationship between survival outcome (OS and DSS) and dichotomized protein expression of CCC-associated biomarkers (a-t). Patients with ARPC2-, KCTD10-, SMYD2-, and TRIO-positive expression revealed significantly shorter OS and DSS. Moreover, positive expression of GNB1, NUP155, RPL13A and SETD3 showed a significant association with OS. Positive CCT5 protein expression was associated with longer DSS, and RPL37-positive protein expression correlated with both longer OS and DSS. The x-axis depict days after initial diagnosis, and the y-axis depict OS (left panel) or DSS (right panel).

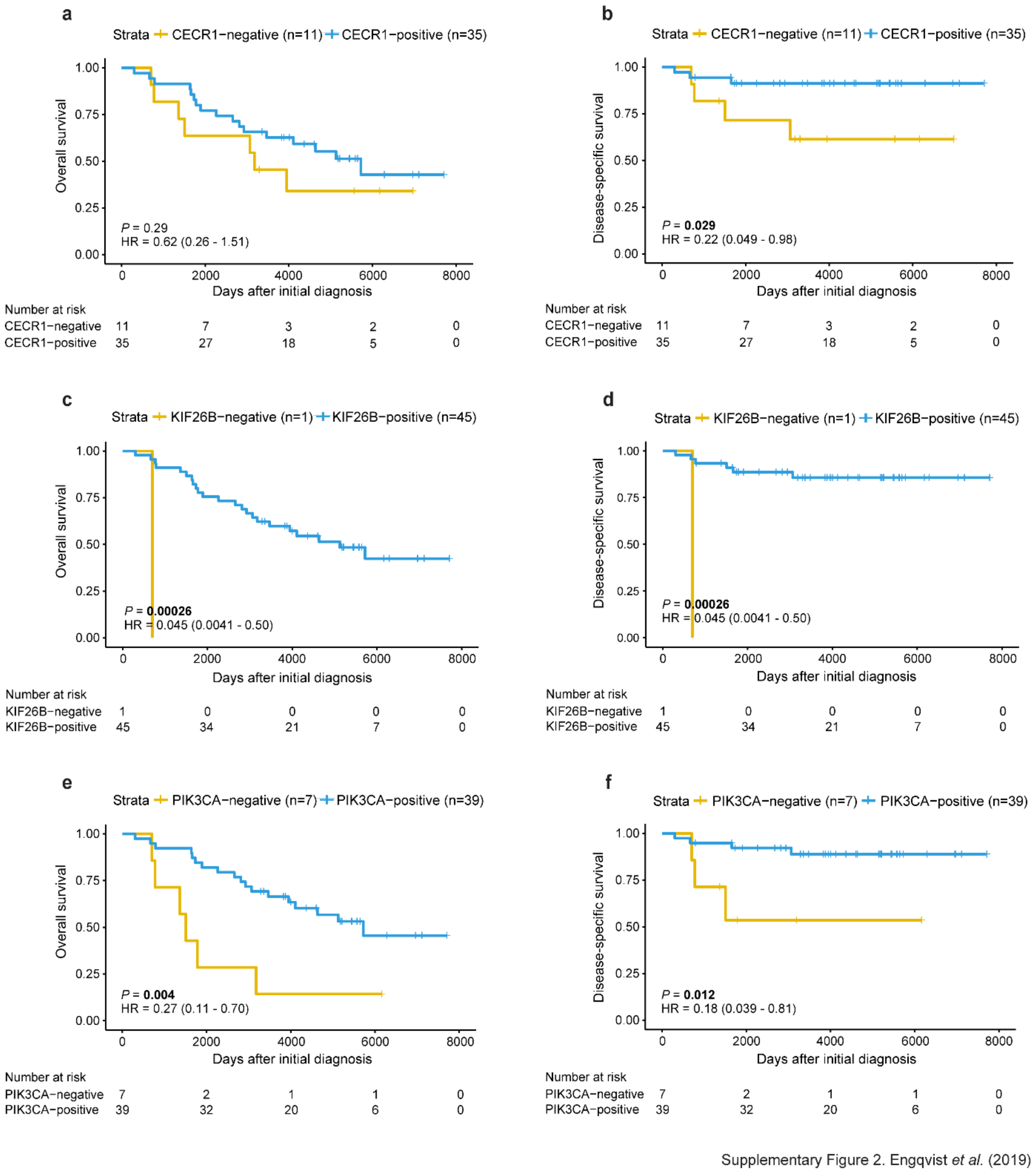

Supplementary Figure 2: Kaplan-Meier plots for the biomarkers related to EC. Kaplan-Meier survival plots illustrating the association between OS (left column) and DSS (right column) with dichotomized protein expression for EC-related biomarkers (a-f). CECR1-positive protein expression associated with longer DSS, and positive protein expression of KIF26B and PIK3CA were related with both longer OS and DSS. The x-axis depict days after initial diagnosis and the y-axis depict survival outcome (OS or DSS).

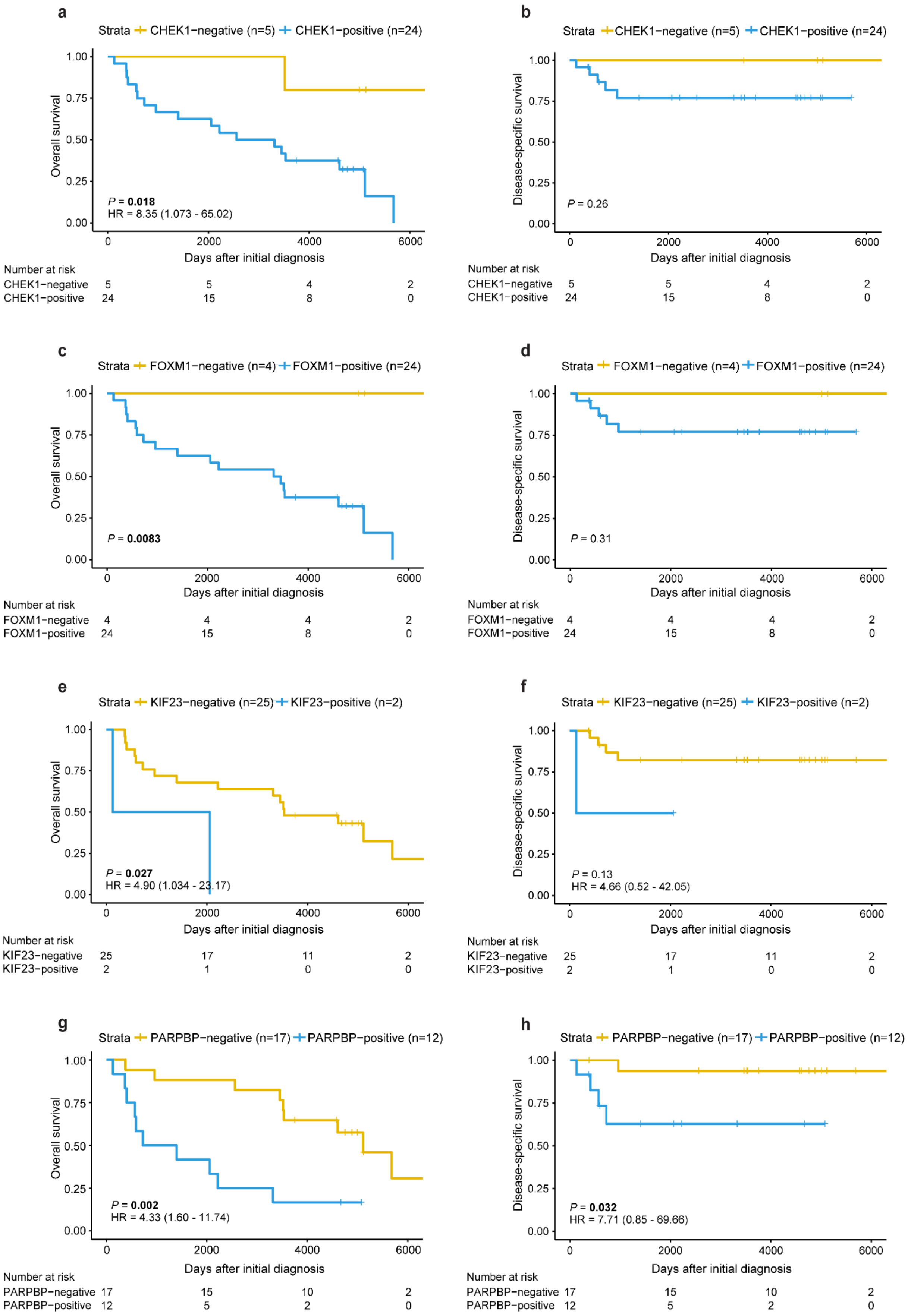

Supplementary Figure 3. Engqvist *et al.* (2019)

Supplementary Figure 3: Kaplan-Meier survival analysis for MC-associated biomarkers. Kaplan-Meier plots revealing an association between dichotomized protein expression of biomarkers related to MC with survival outcome (left panel OS, right panel DSS). CHEK1-, FOXM1-, KIF23- and PARPBP-positive expression related to shorter OS, and PARPBP-positive expression further correlated with shorter DSS. The x-axis depict days after initial diagnosis and the y-axis depict OS or DSS.

Protein	Hazard ratio (95% CI)	P-value
ARPC2		
OS	3.47 (1.41-8.53)	0.0043
DSS	3.21 (1.16-8.91)	0.019
CECR1		
OS	0.62 (0.26-1.51)	0.29
DSS	0.22 (0.049-0.98)	0.029
CCT5		
OS	0.51 (0.24-1.08)	0.073
DSS	0.39 (0.16-0.99)	0.04
CHEK1		
OS	8.35 (1.073-65.02)	0.02
GNB1		
OS	2.35 (0.99-5.57)	0.046
DSS	1.67 (0.63-4.40)	0.3
KCTD10		
OS	3.03 (1.34-6.88)	0.0057
DSS	3.54 (1.26-9.91)	0.011
KIF23		
OS	4.90 (1.034-23.17)	0.027
DSS	4.66 (0.52-42.05)	0.13
KIF26B		
OS	0.045 (0.0041-0.50)	0.00026
DSS	0.045 (0.0041-0.50)	0.00026
NUP155		
OS	8.32 (1.72-40.32)	0.0016
DSS	5.24 (0.63-43.51)	0.087
PARPBP		
OS	4.33 (1.60-11.74)	0.002
DSS	7.71 (0.85-69.66)	0.032
PIK3CA		
OS	0.27 (0.11-0.70)	0.004
DSS	0.18 (0.039-0.81)	0.012
RPL13A		
OS	6.69 (0.78-57.25)	0.045
RPL37		
OS	0.12 (0.013-1.054)	0.022
DSS	0.090 (0.0093-0.86)	0.0085
SETD3		
OS	2.49 (0.99-6.26)	0.044
DSS	2.15 (0.71-6.52)	0.16
SMYD2		
OS	16.99 (1.54-187.40)	0.0015
DSS	33.5 (2.095-535.6)	7e-05
TRIO		
OS	3.39 (0.95-12.07)	0.045
DSS	4.58 (1.24-16.95)	0.012

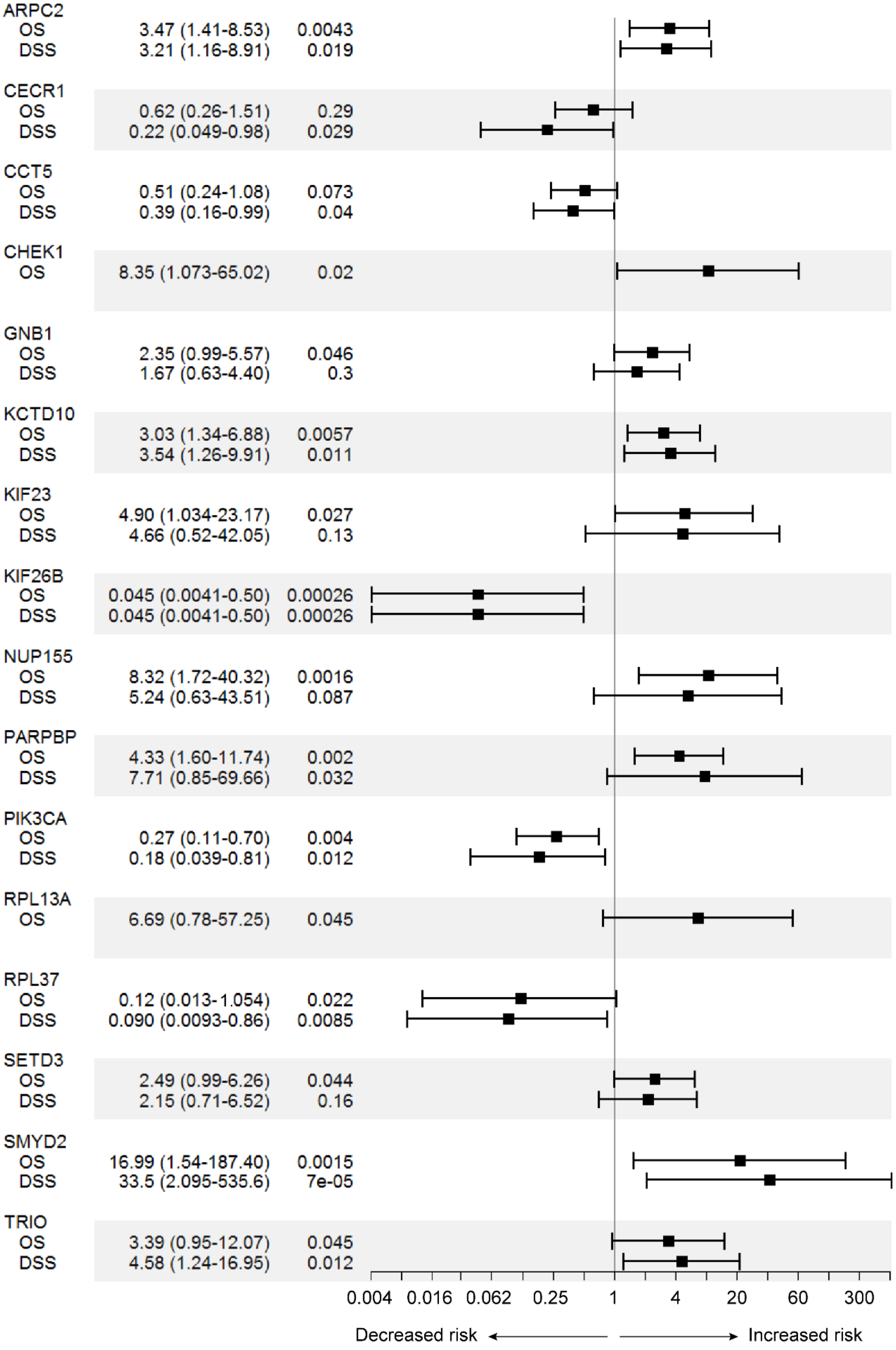

Supplementary Figure 4. Engqvist *et al.* (2019)

Supplementary Figure 4: Forest plot of hazards ratios for mortality for biomarkers linked to clinical outcome. Prognostic biomarkers with HR>1 are coupled to increased risk in view of survival, i.e. patients with positive protein expression of the biomarkers correlate with shorter survival, and prognostic biomarkers with HR<1 are coupled to decreased risk, i.e. patients with positive protein expression correlate with longer survival. FOXM1 has infinite confidence interval and is therefore not included in the plot. The continuous line at 1 indicates no effect on event risk. The x-axis is depicted with a logarithmic scale.

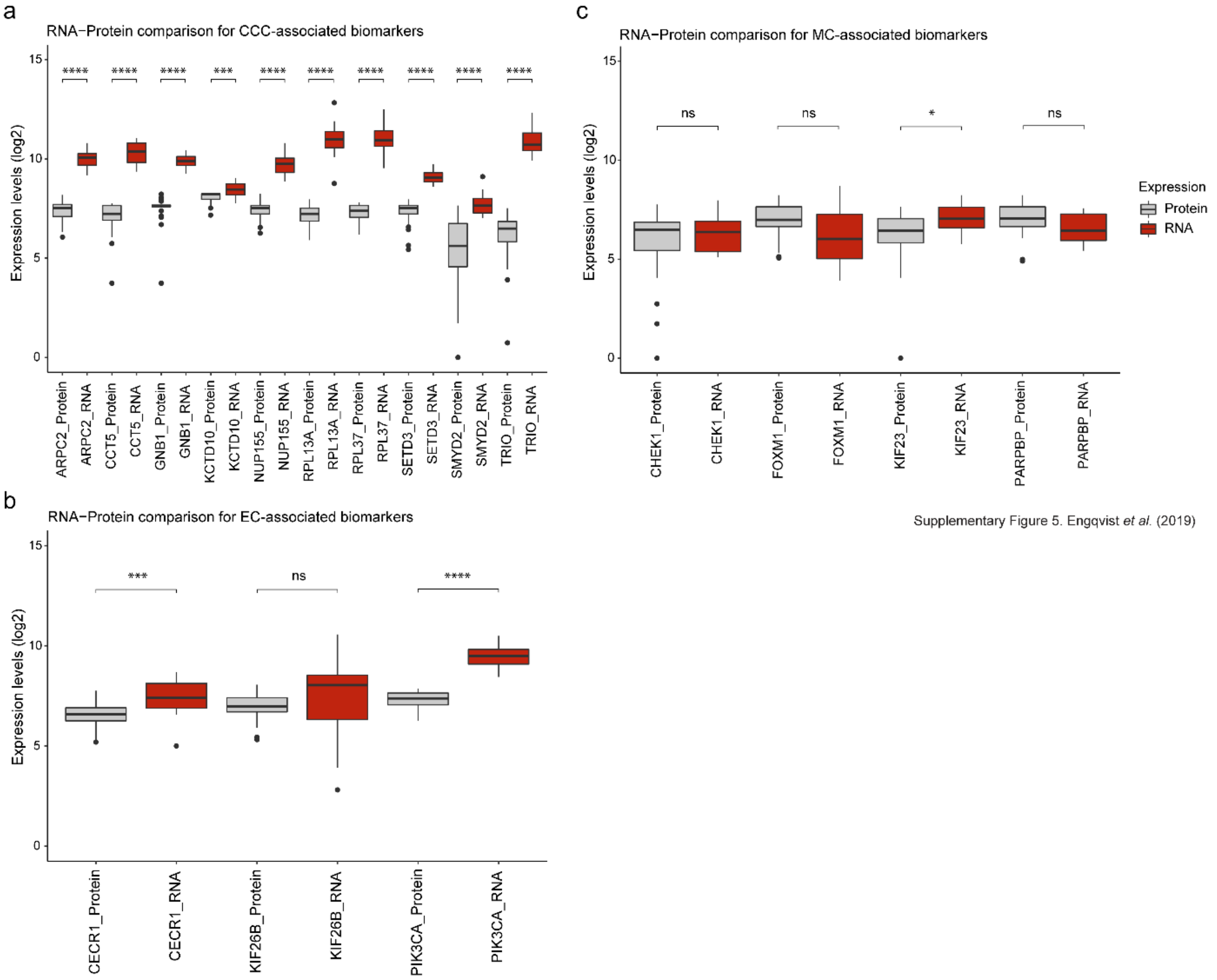

Supplementary Figure 5. RNA-Protein comparison for CCC-, EC-, and MC-associated biomarkers. Box plots illustrating the relationship between protein expression (log2 values of H-score, gray color) and RNA expression (log2 values of raw RNA-seq read counts, red color) for CCC- (a), EC- (b) and MC-related biomarkers (c). Statistical significance was calculated using the Wilcoxon test. P values are shown as: ns = not significant ($P>0.05$); *$P\leq0.05$; **$P\leq0.01$; ***$P\leq0.001$; ****$P\leq0.0001$.

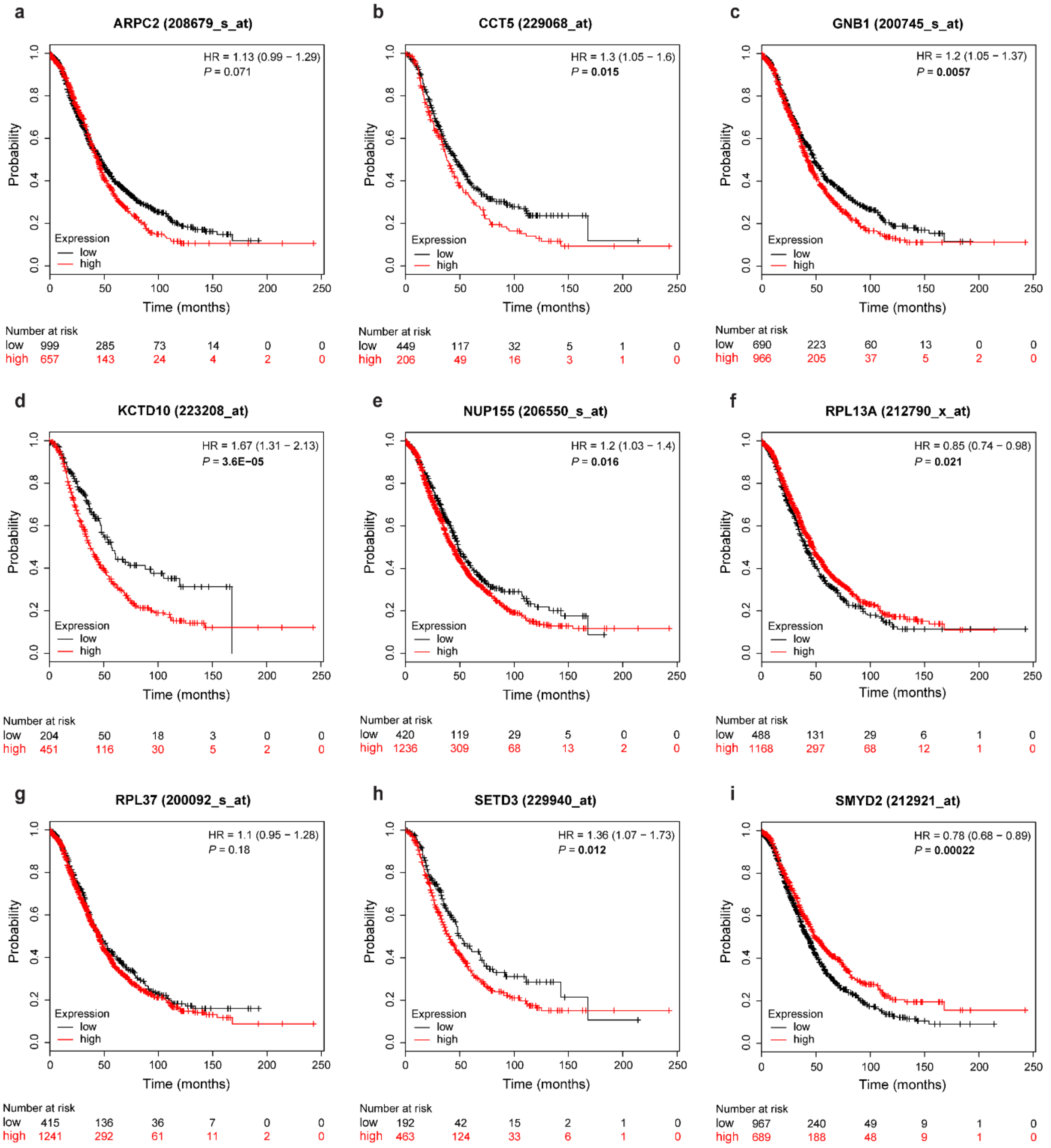

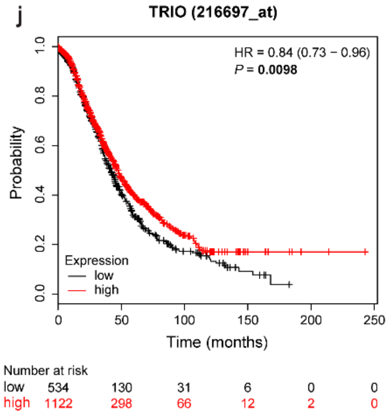

Supplementary Figure 6. Engqvist *et al.* (2019)

Supplementary Figure 6: External Kaplan-Meier survival analysis for CCC-associated biomarkers using gene expression data in KM plotter. KM plotter confirms the prognostic value for 8/10 CCC-associated biomarkers (n=655-1656 HGSC and EC patients), wherein positive expression of CCT5, GNB1, KCTD10, NUP155, and SETD3 were associated with shorter OS, and positive expression of RPL13A, SMYD2 and TRIO were related to longer OS. No significant prognostic value was found for ARPC2 and RPL37 (P values>0.05). Patients with expression levels above the median is shown in red and patients with expression levels below the median is shown in black. Number-at-risk is indicated below the Kaplan-Meier plot.

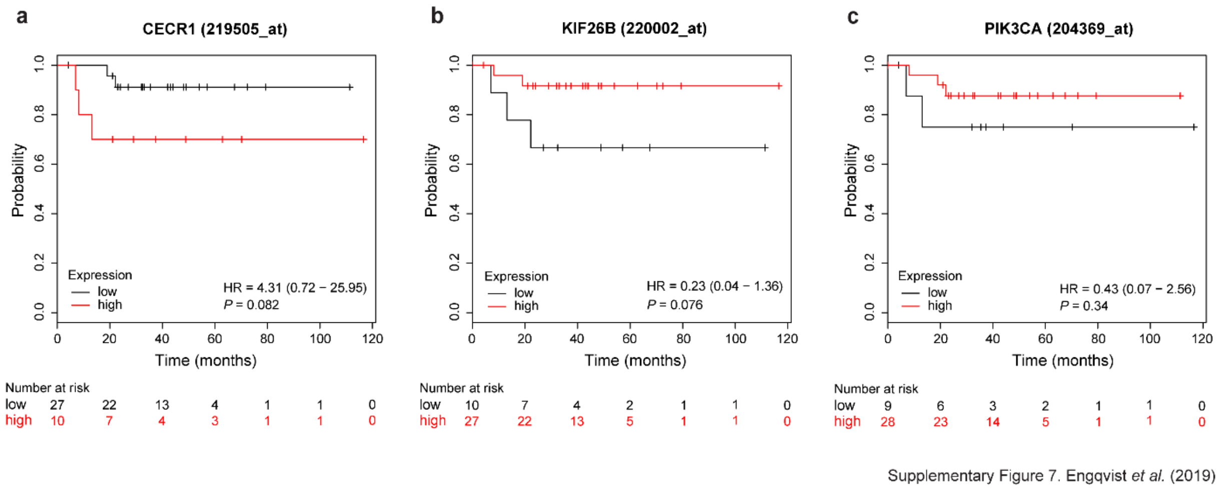

Supplementary Figure 7: External gene expression survival analysis using EC patients in KM Plotter. No significant Kaplan-Meier plots using EC patients (n=37) could be shown for the difference between positive and negative protein expression for the EC-associated biomarkers. For KIF26B and PIK3CA, the same tendency of negative gene expression (black curve) correlated with shorter OS.

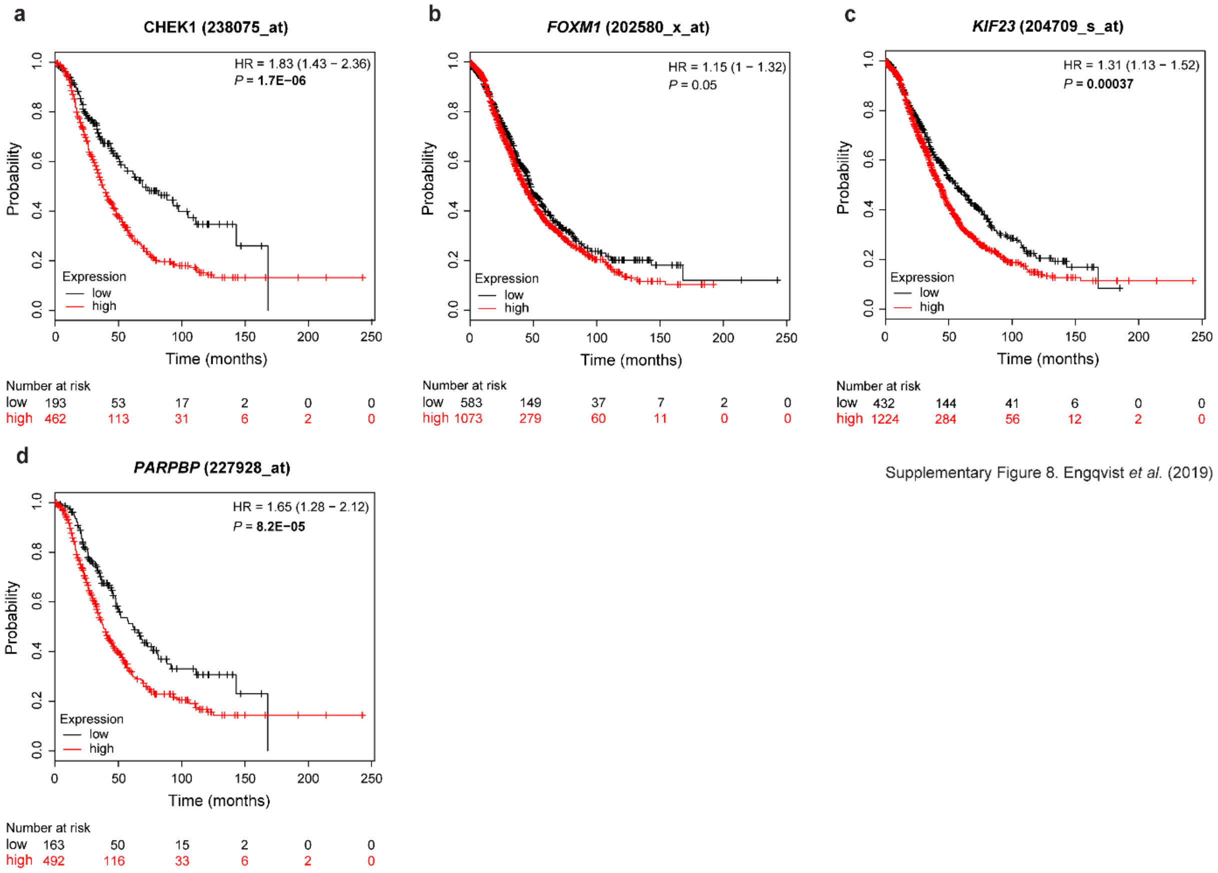

Supplementary Figure 8: Kaplan-Meier survival analysis for MC-associated biomarkers using the external KM plotter ovarian carcinoma dataset. The prognostic value was confirmed for CHEK1, KIF23 and PARPBP on the gene expression level with a correlation between positive gene expression (red curves) and shorter OS. Borderline significance for the same correlation was further found for FOXM1. The Kaplan-Meier analysis was performed in 655 to 1656 HGSC and EC patients.

Supplementary Table 1. Reporting recommendations for tumor marker prognostic studies (REMARK) guidelines.

	Item to be reported
**INTRODUCTION**	
1	*State the marker examined, the study objectives, and any pre-specified hypotheses.*<br>The clinical significance of 29 histotype-specific prognostic biomarkers identified using RNA sequencing (RNA-seq) for early-stage clear-cell (CCC), endometrioid (EC) and mucinous (MC) ovarian carcinoma was validated using immunohistochemistry (IHC) on tissue microarray (TMA).<br>Examined markers:<br>Study cohort: 11 biomarkers associated with CCC (ARPC2, CCT5, DDX24, GNB1, KCTD10, NUP155, RPL13A, RPL37, SETD3, SMYD2, TRIO), 8 with EC (ABCA12, CECR1, ESRRG, KIF26B, MUC15, PDE4DIP, PIK3CA, RIMBP2), and 10 with MC (CENPI, CHEK1, FOXM1, KIF15, KIF23, KNTC1, MTGR1, NSD2, PARPBP, ZDHHC2) (Table 2).<br>Established markers: patient age, stage, CA125, and ploidy.
**MATERIALS AND METHODS**	
Patients	
2	*Describe the characteristics (e.g., disease stage or co-morbidities) of the study patients, including their source and inclusion and exclusion criteria.*<br>The clinicopathological features of the ovarian carcinoma patients (n=112) included in the cohort are described in “Patients and tissue microarray construction” in the Materials and Methods section and in Table 1.
3	*Describe treatments received and how chosen (e.g., randomized or rule-based).*<br>National treatment guidelines with protocols for standard surgery procedures (staging and accurate debulking cytoreductive surgery) were used. The majority of patients (106/112) received adjuvant therapy with chemotherapy (Table 1).
Specimen characteristics	
4	*Describe type of biological material used (including control samples) and methods of preservation and storage.*<br>The patient study cohort comprised 112 early-stage (stage I and II) primary invasive ovarian carcinoma patients (diagnosed between 1994 and 2006) of histotypes clear cell carcinoma (CCC (n=37)), endometrioid carcinoma (EC (n=46)) and mucinous carcinoma (MC (n=29)). TMAs were constructed from full-face formalin-fixed paraffin-embedded (FFPE) specimens obtained from the Departments of Clinical Pathology at hospitals in Western Sweden. One full-face FFPE section from the optimization panel, consisting of 15 full-face FFPE ovarian carcinoma sections representing varying histotypes (HGSC, EC, MC, CCC) and FIGO stages, was chosen as positive control for each immunohistochemical experiment.
Assay methods	
5	*Specify the assay method used and provide (or reference) a detailed protocol, including specific reagents or kits used, quality control procedures, reproducibility assessments, quantitation methods, and scoring and reporting protocols. Specify whether and how assays were performed blinded to the study endpoint.*<br>A pathologists, who was blinded to the survival data, performed the microscopic analysis of immunostained tissue sections. An immunoreactive score (H-score) was determined for each

tumor core based on percentages of stained tumor cells and staining intensities (weak=1, moderate=2, strong=3). The resulting H-score was based on the mean of triplicate TMA cores.

Study design

6 *State the method of case selection, including whether prospective or retrospective and whether stratification or matching (e.g., by stage of disease or age) was used. Specify the time period from which cases were taken, the end of the follow-up period, and the median follow-up time.*

The primary ovarian carcinoma patients (diagnosed between 1994 and 2006, n=112) were retrospectively selected from biobanks at the Departments of Clinical Pathology at hospitals in Western Sweden. Clinicopathological features and survival data (OS/DSS) were obtained from the National Quality Registry at the Regional Cancer Center West (Gothenburg, Sweden) and the Cancer Registry at the National Board of Health and Welfare (Stockholm, Sweden). Patients were chosen for inclusion in the study cohort according to early-stage ovarian carcinomas (stage I and II), overall survival calculated from the date of initial diagnosis to the date of death of any cause, as well as histotype (CCC, EC and MC).

The end of follow-up-period was October 2015 and the median follow-up time was 3436 days.

7 *Precisely define all clinical endpoints examined.*

Overall survival (OS) was defined as the time from initial diagnosis to death from any cause, and disease-specific survival (DSS) was defined as the time from initial diagnosis to ovarian cancer-related death.

8 *List all candidate variables initially examined or considered for inclusion in models.*

Predictive models using multivariable Cox proportional hazard models were used to determine the predictive power (C-index) of the validated biomarkers (n=17; CCC: ARPC2, CCT5, GNB1, KCTD10, NUP155, RPL13A, RPL37, SETD3, SMYD2, TRIO; EC: CECR1, KIF26B, PIK3CA; and MC: CHEK1, FOXM1, KIF23, PARPBP) in combination with established clinical parameters (age, stage, CA125, and ploidy).

9 *Give rationale for sample size; if the study was designed to detect a specified effect size, give the target power and effect size.*

In total, 112 samples were included in the patient cohort comprising 37 CCC, 46 EC and 29 MC samples. The sample size provided sufficient statistical power.

Statistical analysis methods

10 *Specify all statistical methods, including details of any variable selection procedures and other model-building issues, how model assumptions were verified, and how missing data were handled.*

a) *Study cohort selection*
The selection of the 29 promising prognostic biomarkers is described in the "Selection of genes associated with histotype-specific prognosis" in the Materials and Methods section.

b) *Association of marker values/histotypes with clinicopathological characteristics*
Possible confounding factors was evaluated using the R-package tableone (v. 0.9.3) (Table 1, Supplementary Tables 1-3).

c) *Patient survival estimation in relation to protein expression levels*
Survival analysis was performed with Kaplan-Meier curves and compared with log rank tests using survival time (OS/DSS) and dichotomized H-score for positive immunostaining (survival v. 2.40-1 and survminer v. 0.4.3) (Figures 2-4, Supplementary Figures 1-3).

d) *Methods to evaluate the biomarker's univariable and multivariable association with clinical outcome*
Univariable and multivariable Cox proportional hazard models were used to assess the predictive power (C-index) of the CCC- (ARPC2, CCT5, GNB1, KCTD10, NUP155, RPL13A, RPL37, SETD3, SMYD2, TRIO), EC- (CECR1, KIF26B, PIK3CA) and MC-associated biomarkers (CHEK1, FOXM1, KIF23, PARPBP) in view of survival outcome (OS/DSS) when adjusted for established clinical parameters (age, stage, CA125, and ploidy) (Supplementary Tables 4-6).

e) *Comparison between RNA and protein expression*
Box plots comparing protein and RNA expression were generated using the R package ggplot2 (v. 3.1.0) and Kruskal-Wallis test.

f) *External validation*
The web-based Kaplan-Meier plotter tool (KM plotter, http://kmplot.com/analysis/) for ovarian cancer was used to determine the clinical relevance of gene expression for the study cohort in relation to overall survival in an external cohort.

g) *Missing data*
In the survival analysis, patients with missing values (Table 1, "Not available") were excluded.

11 *Clarify how marker values were handled in the analyses; if relevant, describe methods used for cutpoint determination.*

Protein expression levels (H-score values) were correlated with clinical outcome (OS/DSS). Kaplan-Meier plots in X-tile software (v. 3.6.1) was used to dichotomize H-score cutoff values into positive and negative protein expression groups.

RESULTS

Data

12 *Describe the flow of patients through the study, including the number of patients included in each stage of the analysis (a diagram may be helpful) and reasons for dropout. Specifically, both overall and for each subgroup extensively examined report the numbers of patients and the number of events.*

The Tables 1 and 2 describe the number of patients used in the different analyses.

13 *Report distributions of basic demographic characteristics (at least age and sex), standard (disease-specific) prognostic variables, and tumour marker, including numbers of missing values.*

The clinicopathological features including numbers of missing values for the 112 patients are listed in Table 1.

Analysis and presentation

14 *Show the relation of the marker to standard prognostic variables.*

Figures 2-4 and Supplementary Tables 1-3 show the relationship between prognostic markers and established clinicopathological data.

15 *Present univariable analyses showing the relation between the marker and outcome, with the estimated effect (e.g., hazard ratio and survival probability). Preferably provide similar analyses for all other variables being analysed. For the effect of a tumour marker on a time-to-event outcome, a Kaplan-Meier plot is recommended.*

Kaplan-Meier plots and univariable analyses including hazard ratio, *P* value and C-index for the CCC-, EC- and MC-associated biomarkers, are shown in Figures 2-4 and Supplementary Tables 4-6.

16	*For key multivariable analyses, report estimated effects (e.g., hazard ratio) with confidence intervals for the marker and, at least for the final model, all other variables in the model.*<br><br>Multivariable analyses including hazard ratio, *P* value and C-index for the CCC-, EC- and MC-associated biomarkers are shown in Supplementary Tables 4-6.
17	*Among reported results, provide estimated effects with confidence intervals from an analysis in which the marker and standard prognostic variables are included, regardless of their statistical significance.*<br><br>Univariable and multivariable analyses including established clinical parameters (age, stage, CA125, and ploidy) are described in Supplementary Tables 4-6.
18	*If done, report results of further investigations, such as checking assumptions, sensitivity analyses, and internal validation.*<br><br>The external validation results are described in the Results section.
**DISCUSSION**	
19	*Interpret the results in the context of the pre-specified hypotheses and other relevant studies; include a discussion of limitations of the study.*<br><br>The study results were interpreted in the context of pre-specified hypotheses and other relevant studies in the Discussion section.
20	*Discuss implications for future research and clinical value.*<br><br>Further investigation using larger patient cohorts is needed, perhaps with patients from different regions and/or countries, to further validate our conclusions. Moreover, *in vitro* and *in vivo* models could further validate the clinical and biological significance of the studied biomarkers in CCC, EC and MC ovarian carcinoma histotypes.

Supplementary Table 2. Distribution of clinicopathological characteristics in relation to protein expression of CCC-associated biomarkers.

	ARPC2 expression, n (%)		*P* value	CCT5 expression, n (%)		*P* value	GNB1 expression, n (%)		*P* value	KCTD10 expression, n (%)		*P* value	NUP155 expression, n (%)		*P* value
	Negative (n=28)	Positive (n=9)		Negative (n=19)	Positive (n=18)		Negative (n=12)	Positive (n=25)		Negative (n=15)	Positive (n=22)		Negative (n=35)	Positive (n=2)	
**Patient age**			**0.012**						0.291			0.89			0.63
Mean	62.3	73.2		66.2	63.6	0.50	63.6	65.6		64.6	65.1		65.1	61	
Range	42-84	55-84		42-84	49-84		42-79	49-84		49-84	42-84		42-84	55-67	
**Stage**			1.00						0.67			1.00			0.73
I	23 (82)	8 (89)		3 (16)	3 (17)	1.00	11 (92)	25 (80)		13 (87)	18 (82)		30 (86)	1 (50)	
II	5 (18)	1 (11)		16 (84)	15 (83)		1 (8)	5 (20)		2 (13)	4 (18)		5 (14)	1 (50)	
**CA125**			0.39			0.66			0.39			0.82			0.21
<35	10 (36)	4 (44)		6 (32)	8 (44)		5 (42)	9 (36)		5 (33)	9 (41)		14 (40)	0 (0)	
35-65	5 (18)	3 (33)		5 (26)	3 (17)		1 (8)	7 (28)		3 (20)	5 (23)		8 (23)	0 (0)	
>65	13 (46)	2 (22)		8 (42)	7 (39)		6 (50)	9 (36)		7 (47)	8 (36)		13 (37)	2 (100)	
**Ploidy**			0.68			0.32			0.78			0.59			0.78
Near diploid	4 (14)	1 (11)		3 (16)	2 (11)		2 (17)	3 (12)		1 (7)	4 (18)		5 (14)	0 (0)	
Aneuploid	23 (82)	7 (78)		14 (74)	16 (89)		9 (75)	21 (84)		13 (87)	17 (77)		28 (80)	2 (100)	
Not available	1 (4)	1 (11)		2 (11)	0 (0)		1 (8)	1 (4)		1 (7)	1 (5)		2 (6)	0 (0)	
**Chemotherapy**			NA			NA			NA			NA			NA
Yes	28 (100)	9 (100)		19 (100)	18 (100)		12 (100)	25 (100)		15 (100)	22 (100)		35 (100)	2 (100)	
No	0 (0)	0 (0)		0 (0)	0 (0)		0 (0)	0 (0)		0 (0)	0 (0)		0 (0)	0 (0)	

	RPL13A expression, n (%)		*P* value	RPL37 expression, n (%)		*P* value	SETD3 expression, n (%)		*P* value	SMYD2 expression, n (%)		*P* value	TRIO expression, n (%)		*P* value
	Negative (n=36)	Positive (n=1)		Negative (n=1)	Positive (n=36)		Negative (n=31)	Positive (n=6)		Negative (n=35)	Positive (n=1)		Negative (n=34)	Positive (n=3)	
**Patient age**			NA			NA			0.082			NA			0.21
Mean	64.7	74.0		63.0	65.0		63.5	72.5		63.9	80.0		65.7	56.7	
Range	42-84	-		-	42-84		42-84	56-81		42-84	-		42-84	51-62	
**Stage**			1.00			1.00			0.57			1.00			0.98
I	30 (83)	1 (100)		1 (100)	30 (83)		25 (81)	6 (100)		29 (83)	1 (100)		29 (85)	2 (67)	
II	6 (17)	0 (0)		0 (0)	6 (17)		6 (19)	0 (0)		6 (17)	0 (0)		5 (15)	1 (33)	
**CA125**			0.43			0.47			0.16			0.49			0.53
<35	13 (36)	1 (100)		0 (0)	14 (39)		12 (39)	2 (33)		13 (37)	0 (0)		13 (38)	1 (33)	
35-65	8 (22)	0 (0)		0 (0)	8 (22)		5 (16)	3 (50)		8 (23)	0 (0)		8 (24)	0 (0)	
>65	15 (42)	0 (0)		1 (100)	14 (39)		14 (45)	1 (17)		14 (40)	1 (100)		13 (38)	2 (67)	
**Ploidy**			0.89			0.89			**0.016**			0.88			0.68
Near diploid	5 (14)	0 (0)		0 (0)	5 (14)		2 (7)	3 (50)		5 (14)	0 (0)		5 (15)	0 (0)	
Aneuploid	29 (81)	1 (100)		1 (100)	29 (81)		27 (87)	3 (50)		28 (80)	1 (100)		27 (79)	3 (100)	
Not available	2 (6)	0 (0)		0 (0)	2 (6)		2 (7)	0 (0)		2 (6)	0 (0)		2 (6)	0 (0)	
**Chemotherapy**			NA			NA			NA			NA			NA
Yes	36 (100)	1 (100)		1 (100)	36 (100)		31 (100)	6 (100)		35 (100)	1 (100)		34 (100)	3 (100)	
No	0 (0)	0 (0)		0 (0)	0 (0)		0 (0)	0 (0)		0 (0)	0 (0)		0 (0)	0 (0)	

Supplementary Table 3. Distribution of clinicopathological characteristics in relation to protein expression of EC-associated biomarkers.

	CECR1 expression, n (%)		*P* value	KIF26B expression, n (%)		*P* value	PIK3CA expression, n (%)		*P* value
	Negative (n=11)	Positive (n=35)		Negative (n=1)	Positive (n=45)		Negative (n=7)	Positive (n=39)	
**Patient age**			0.82			NA			0.25
Mean	61.0	62.2		54.0	62.1		56.0	63.0	
Range	46-79	25-83		-	25-83		48-68	25-83	
**Stage**			0.39			1.00			0.22
I	6 (55)	26 (74)		1 (100)	31 (69)		3 (43)	29 (74)	
II	5 (46)	9 (26)		0 (0)	14 (31)		4 (57)	10 (26)	
**Tumor grade**			0.59			0.70			0.63
FIGO grade I	2 (18)	9 (26)		0 (0)	11 (24)		1 (14)	10 (26)	
FIGO grade II	6 (55)	21 (60)		1 (100)	26 (58)		4 (57)	23 (59)	
FIGO grade III	3 (27)	5 (14)		0 (0)	8 (18)		2 (29)	6 (15)	
**CA125**			0.82			0.84			0.60
<35	3 (27)	10 (29)		0 (0)	13 (29)		2 (29)	11 (28)	
35-65	1 (9)	6 (17)		0 (0)	7 (16)		0 (0)	7 (18)	
>65	7 (64)	18 (51)		1 (100)	24 (53)		5 (71)	20 (51)	
Not available	0 (0)	1 (3)		0 (0)	1 (2)		0 (0)	1 (3)	
**Ploidy**			0.54			0.42			0.74
Near diploid	5 (46)	12 (34)		1 (100)	16 (36)		3 (43)	14 (36)	
Aneuploid	6 (55)	20 (57)		0 (0)	26 (58)		4 (57)	22 (56)	
Not available	0 (0)	3 (9)		0 (0)	3 (7)		0 (0)	3 (8)	
**Chemotherapy**			1.00			1.00			0.87
Yes	10 (91)	32 (91)		1 (100)	41 (91)		7 (100)	35 (90)	
No	1 (9)	3 (9)		0 (0)	4 (9)		0 (0)	4 (10)	

Supplementary Table 4. Distribution of clinicopathological characteristics in relation to protein expression of MC-associated biomarkers.

	CHEK1 expression, n (%)			FOXM1 expression, n (%)			KIF23 expression, n (%)			PARPBP expression, n (%)		
	Negative (n=5)	Positive (n=24)	***P* value**	Negative (n=4)	Positive (n=24)	***P* value**	Negative (n=25)	Positive (n=2)	***P* value**	Negative (n=17)	Positive (n=12)	***P* value**
**Patient age**			0.85			0.95			0.97			0.80
Mean	61.4	60.0		60.0	59.5		60.1	60.5		59.7	61.1	
Range	46-77	30-82		46-77	30-82		30-82	57-64		30-82	43-80	
**Stage**			0.74			1.00			1.00			0.73
I	3 (60)	19 (79)		3 (75)	19 (79)		19 (76)	2 (100)		12 (71)	10 (83)	
II	2 (40)	5 (21)		1 (25)	5 (21)		6 (24)	0 (0)		5 (29)	2 (17)	
**CA125**			0.53			0.24			0.21			0.94
<35	1 (20)	9 (38)		1 (25)	9 (38)		9 (36)	0 (0)		6 (35)	4 (33)	
35-65	1 (20)	7 (29)		0 (0)	7 (29)		7 (28)	0 (0)		5 (29)	3 (25)	
>65	3 (60)	8 (33)		3 (75)	8 (33)		9 (36)	2 (100)		6 (35)	5 (42)	
**Ploidy**			0.74			0.57			0.64			0.30
Near diploid	1 (20)	6 (25)		1 (25)	5 (21)		5 (20)	0 (0)		4 (24)	3 (25)	
Aneuploid	3 (60)	16 (67)		2 (50)	17 (71)		17 (68)	2 (100)		10 (58)	9 (75)	
Not available	1 (20)	2 (8)		1 (25)	2 (8)		3 (12)	0 (0)		3 (18)	0 (0)	
**Chemotherapy**			1.00			1.00			1.00			1.00
Yes	5 (100)	22 (92)		4 (100)	22 (92)		23 (92)	2 (100)		16 (94)	11 (92)	
No	0 (0)	2 (8)		0 (0)	2 (8)		2 (8)	0 (0)		1 (6)	1 (8)	

Supplementary Table 5: Univariable and multivariable survival analysis for CCC-associated biomarkers.

	Overall survival			Disease-specific survival		
	HR (95% CI)	*P* value	C-index	HR (95% CI)	*P* value	C-index
**Univariable analysis**						
Patient age	1.021	0.20	0.57	1.01	0.50	0.55
Stage	1.04	0.90	0.50	0.73	0.70	0.53
CA125	-	0.60	0.56	-	0.20	0.60
Ploidy	-	0.20	0.54	-	0.10	0.56
ARPC2	**3.47**	**0.0043**	**0.61**	**3.21**	**0.019**	**0.60**
CCT5	0.51	0.073	0.60	**0.39**	**0.040**	**0.62**
GNB1	**2.35**	**0.046**	**0.61**	1.67	0.30	0.56
KCTD10	**3.03**	**0.0057**	**0.63**	**3.54**	**0.011**	**0.65**
NUP155	**8.32**	**0.0016**	**0.55**	5.24	0.087	0.53
RPL13A	**6.69**	**0.045**	**0.53**	-	0.73	0.51
RPL37	**0.12**	**0.022**	**0.53**	**0.090**	**0.0085**	**0.53**
SETD3	**2.49**	**0.044**	**0.57**	2.15	0.16	0.56
SMYD2	**16.99**	**0.0015**	**0.53**	**33.50**	**0.000070**	**0.54**
TRIO	**3.39**	**0.045**	**0.55**	**4.58**	**0.012**	**0.57**
**Multivariable analysis**						
ARPC2	**3.36**	**0.033**	**0.67**	2.75	0.110	0.69
CCT5	0.62	0.27	0.64	0.44	0.12	0.69
GNB1	**2.52**	**0.043**	**0.67**	1.58	0.37	0.66
KCTD10	**5.96**	**0.00065**	**0.74**	**7.21**	**0.0016**	**0.77**
NUP155	**12.17**	**0.0049**	**0.67**	7.28	0.088	0.66
RPL13A	**10.68**	**0.043**	**0.65**	2.42E-7	1.00	0.65
RPL37	0.11	0.056	0.64	**0.075**	**0.034**	**0.66**
SETD3	**8.31**	**0.011**	**0.67**	5.37	0.11	0.66
SMYD2	12.35	0.056	0.64	**36.73**	**0.02**	**0.67**
TRIO	3.46	0.13	0.64	4.83	0.074	0.66

Supplementary Table 6: Univariable and multivariable survival analysis for EC-associated biomarkers.

	Overall survival			Disease-specific survival		
	**HR (95% CI)**	***P* value**	**C-index**	**HR (95% CI)**	***P* value**	**C-index**
**Univariable analysis**						
Patient age	**1.045**	**0.009**	**0.67**	1.0016	0.90	0.51
Stage	**0.69**	**0.005**	**0.65**	1.48	0.70	0.58
CA125	-	0.40	0.59	-	0.60	0.62
Ploidy	-	0.10	0.60	-	0.60	0.58
CECR1	0.62	0.29	0.54	**0.22**	**0.029**	**0.68**
KIF26B	**0.045**	**0.00026**	**0.53**	**0.045**	**0.00026**	**0.57**
PIK3CA	**0.27**	**0.0040**	**0.59**	**0.18**	**0.012**	**0.66**
**Multivariable analysis**						
CECR1	0.58	0.25	0.72	0.18	0.057	0.81
KIF26B	**0.019**	**0.0012**	**0.74**	**0.021**	**0.0016**	**0.80**
PIK3CA	**0.08**	**0.00019**	**0.79**	0.17	0.066	0.79

Supplementary Table 7: Univariable and multivariable survival analysis for MC-associated biomarkers.

	Overall survival			Disease-specific survival		
	HR (95% CI)	*P* value	C-index	HR (95% CI)	*P* value	C-index
**Univariable analysis**						
Patient age	1.022	0.20	0.59	0.98	0.5	0.60
Stage	1.074	0.90	0.50	4.57	0.070	0.68
CA125	-	1.00	0.490	-	0.40	0.64
Ploidy	-	0.60	0.57	-	0.20	0.69
CHEK1	**8.35**	**0.018**	**0.62**	-	0.26	0.60
FOXM1	**-**	**0.0083**	**0.63**	-	0.31	0.58
KIF23	**4.90**	**0.027**	**0.56**	4.66	0.13	0.57
PARPBP	**4.33**	**0.002**	**0.65**	**7.71**	**0.032**	**0.73**
**Multivariable analysis**						
CHEK1	**19.68**	**0.015**	**0.76**	1.30E9	1.00	0.90
FOXM1	1.88E9	1.00	0.77	5.38E8	1.00	0.88
KIF23	**8.78**	**0.025**	**0.67**	4.45	0.32	0.89
PARPBP	**8.30**	**0.0011**	**0.74**	35.67	0.077	0.94

Supplementary Table 8: External validation using KM plotter. Evaluation of for the prognostic value of the CCC-, EC- and MC-associated biomarkers for different KM plotter probe IDs.

Gene symbol	Probe 1		Probe 2		Probe 3		Probe 4		Probe 5		Probe 6		Probe 7		Probe 8		Probe 9		Probe 10	
	**Probe**	***P*-value**	**Probe 2**	***P*-value**	**Probe 3**	***P*-value**	**Probe 4**	***P*-value**	**Probe 5**	***P*-value**	**Probe 6**	***P*-value**	**Probe 7**	***P*-value**	**Probe 8**	***P*-value**	**Probe 9**	***P*-value**	**Probe 10**	***P*-value**
ABCA12	215465_at	**2.60E-05**																		
ARPC2	213513_x_at	0.088	207988_s_at	0.2	208679_s_at	0.071														
CCT5	229068_at	**0.015**	208696_at	0.25																
CECR1	219505_at	**0.011**																		
CENPI	207590_s_at	**0.033**	214804_at	0.099																
CHEK1	238075_at	**1.7E-06**	205393_s_at	**0.024**	205394_at	**0.0065**														
DDX24	200694_s_at	**0.016**	200702_s_at	0.2																
ESRRG	207981_s_at	0.23	209966_x_at	0.33																
FOXM1	202580_x_at	0.05																		
GNB1	200746_s_at	**0.012**	200744_s_at	**0.044**	200745_s_at	**0.0057**														
KCTD10	226518_at	**0.011**	223208_at	**3.60E-05**																
KIF15	219306_at	**0.0087**																		
KIF23	204709_s_at	**3.70E-04**																		
KIF26B	220002_at	**0.0021**	1555164_at	0.078																
KNTC1	206316_s_at	**0.0023**																		
MTGR1	207625_s_at	**0.011**	209144_s_at	0.06	209145_s_at	0.12														
MUC15	227238_at	0.17	227241_at	0.16																
NSD2	209052_s_at	0.1	209053_s_at	0.18	209054_s_at	0.19														
NUP155	206550_s_at	**0.016**																		
PARPBP	227928_at	**8.2E-05**																		
PDE4DIP	210305_at	**0.034**	211751_at	0.074	212390_at	**0.0054**	212392_s_	0.21	213388_at	0.28	214099_s_at	0.19	214129_at	0.23	214130_s_	0.097	205872_x_at	0.23	209700_x_at	0.071
PIK3CA	204369_at	0.11																		
RIMBP2	214811_at	**9.00E-05**																		
RPL13A	200715_x_at	**0.048**	212790_x_at	**0.021**	200716_x_at	0.082	210646_x_	0.11	211942_x_at	0.21										
RPL37	200092_s_at	0.18																		
SETD3	229940_at	**0.012**	212465_at	0.19																
SMYD2	212921_at	**2.20E-04**	212922_s_at	0.17																
TRIO	216697_at	**0.0098**	209010_s_at	**0.045**	209011_at	0.28	209012_at	0.3	209013_x_at	0.09	216700_at	0.2	208178_x_at	0.3						
ZDHHC2	222730_s_at	**0.014**	222731_at	**0.026**	244799_at	**0.016**														
